# A Systematic Review on the Hazard Assessment of Amorphous Silica Based on the Literature From 2013 to 2018

**DOI:** 10.3389/fpubh.2022.902893

**Published:** 2022-06-15

**Authors:** Harald F. Krug

**Affiliations:** ^1^NanoCASE GmbH, Engelburg, Switzerland; ^2^Empa—Swiss Federal Laboratories for Science and Materials Technology, St. Gallen, Switzerland; ^3^Faculty of Medicine, University of Berne, Bern, Switzerland

**Keywords:** nanotoxicology, synthetic amorphous silica, hazard assessment, study quality, nanostructured particles, nanomaterials, database

## Abstract

**Background:**

Nanomaterials are suspected of causing health problems, as published studies on nanotoxicology indicate. On the other hand, some of these materials, such as nanostructured pyrogenic and precipitated synthetic amorphous silica (SAS) and silica gel, have been used for decades without safety concerns in industrial, commercial, and consumer applications. However, in addition to many *in vivo* and *in vitro* studies that have failed to demonstrate the intrinsic toxicity of SAS, articles periodically emerge, in which biological effects of concern have been described. Even though most of these studies do not meet high-quality standards and do not always use equivalent test materials or standardized test systems, the results often trigger substance re-evaluation. To put the results into perspective, an extensive literature study was carried out and an example of amorphous silica will be used to try to unravel the reliability from the unreliable results.

**Methods:**

A systematic search of studies on nanotoxicological effects has been performed covering the years 2013 to 2018. The identified studies have been evaluated for their quality regarding material and method details, and the data have been curated and put into a data collection. This review deals only with investigations on amorphous silica.

**Results:**

Of 18,162 publications 1,217 have been selected with direct reference to experiments with synthetically produced amorphous silica materials. The assessment of these studies based on defined criteria leads to a further reduction to 316 studies, which have been included in this systematic review. Screening for quality with well-defined quantitative criteria following the GUIDE nano concept reveals only 27.3% has acceptable quality. Overall, the *in vitro* and *in vivo* data showed low or no toxicity of amorphous silica. The data shown do not support the hypothesis of dependency of biological effects on the primary particle size of the tested materials.

**Conclusion:**

This review demonstrates the relatively low quality of most studies published on nanotoxicological issues in the case of amorphous silica. Moreover, mechanistic studies are often passed off or considered toxicological studies. In general, standardized methods or the Organization for Economic Cooperation and Development (OECD) guidelines are rarely used for toxicological experiments. As a result, the significance of the published data is usually weak and must be reevaluated carefully before using them for regulatory purposes.

## Introduction

The development of innovative materials, such as nanomaterials, is strongly upward and will continue to rise in the future. Even if not all materials find their way to the market, an enormous number of new nanomaterials are being researched in the world's laboratories. This is the main reason why the number of published studies on new materials is also steadily increasing. Around 5,000 published articles per year on “nanotoxicology” (1) make it almost impossible to consider all results when it comes to risk analysis for the use of nanomaterials in our daily life. Moreover, many studies are still being conducted on nanostructured materials that have been on the market for decades, such as synthetic amorphous silica (SAS) and nanoforms of titanium dioxide. The reason for this development is still the working hypothesis right from the beginning: the smaller the nanomaterials, the higher their potency to induce adverse effects. The basis for this working hypothesis lies in the following assumptions that have been described elsewhere (2): specific physicochemical properties, such as smallness lead to better transport in biological systems; a much larger specific surface area compared to larger particles induces higher reactivity and specific material modifications, e.g., one-, two-, or three-dimensional materials add some specific aspects of toxicology. The question remains to what extent these smallest particles can fulfill this paradigm and trigger size-dependent toxicological effects (3), and whether this also applies to materials that have been on the market for more than eight decades, such as synthetic amorphous silica (4, 5), or it is still simply dose-dependent (6).

Unfortunately, the situation in hazard and risk assessment of nanomaterials is not as clear as expected when considering the huge amount of publications during the last two decades of research based on many funding programs at the national or international level (7) resulting in a multitude of studies and publications (compare^1^). As mentioned above, actually, there exist more than 50,000 articles on the biological effects of nanomaterials (8). One could imagine that this might lead to higher safety at workplaces or for the consumer or the environment. Unfortunately, this is not the case, as we have the obscure situation that many publications on the topic “nanotoxicology” do not deliver relevant toxicological data (9–11), and there is still no consensus on the toxicity of nanomaterials (12).

Difficulties in assessing toxicological studies on nanomaterials have been described in detail 10 years ago by Card et al. (13), and they found that 75% of published studies have deficiencies in their study design and are not fully reliable for risk assessment or regulatory purposes. At the same time in 2010, we described first a criteria catalog for assessing the minimum study quality for our informative website of the DaNa-project^2^ (download of the criteria catalog^3^) and Card and Magnuson (14) published their concept for quality scoring of toxicological studies for nanomaterials. This resulted in the demand for reliable and comparable results based on the principle of “stable stool,” in this case with four legs: (i) validation, (ii) traceability, (iii) quality-management system, and (iv) measurement uncertainty (15). Without adherence to these basic principles, it is difficult to classify studies as reliable for regulatory-accepted risk assessment. As the quality of published toxicological data has not increased over the last 10 years (1), the question may be allowed: what is the reason for this lack of reliability? Many scientists and working groups have been looking for what could be the cause that statements of toxicological studies are, in many cases, not very reliable and describe various pitfalls and flaws in nanotoxicology. In general, there exist many interferences of nanomaterials with the used assay systems for human toxicological (16, 17) as well as ecotoxicological studies (18). Moreover, specific interactions in testing by flow cytometry (19) using the comet assay (20, 21), investigating immunomodulatory effects (22), or simply using common cytotoxicity assays (23) are often not respected. Another very important point is the possible contamination of nanomaterials with bacterial endotoxins (24), which induce false-positive inflammatory effects (25, 26). Additionally, investigated materials, such as SAS, may interfere with the determination of endotoxin contamination in the common LAL test and cause wrong results (27).

These and many more mistakes can be committed in experiments with nanomaterials when investigating their toxicological potential (28). The above-mentioned pitfalls and contradictions specifically in hazard assessment of nanomaterials have been the starting point for a project, which, in the end, collected more than 25,000 citations. Nearly 9,000 studies have been evaluated further, and this resulted in more than 6,500 datasets on various nanomaterials. This data collection is the basis for an extensive literature study on amorphous silica that is shown here.

## Methods

### Search Strategy

Several online libraries, such as PubMed^®^, Google Scholar, and Isi Web of Knowledge, were searched from 2013 to 2018, with a search profile to find all publications on the toxicology of nanomaterials (for the specific search profile, refer to Supplementary Material). The keywords were directly related to this topic, such as: “nanotox,^*^” “nanotube,^*^” and “toxic;^*^” “nanoparticle^*^ and toxic^*^;” “nanomat^*^ and toxic^*^;” and some more combined with the year of appearance. The asterisk represents a wildcard within the search terms. With these search terms, between 3,000 and 5,000 publications have been found per year (1).

### Selection of Studies on Amorphous Silica

**Figure 1:**
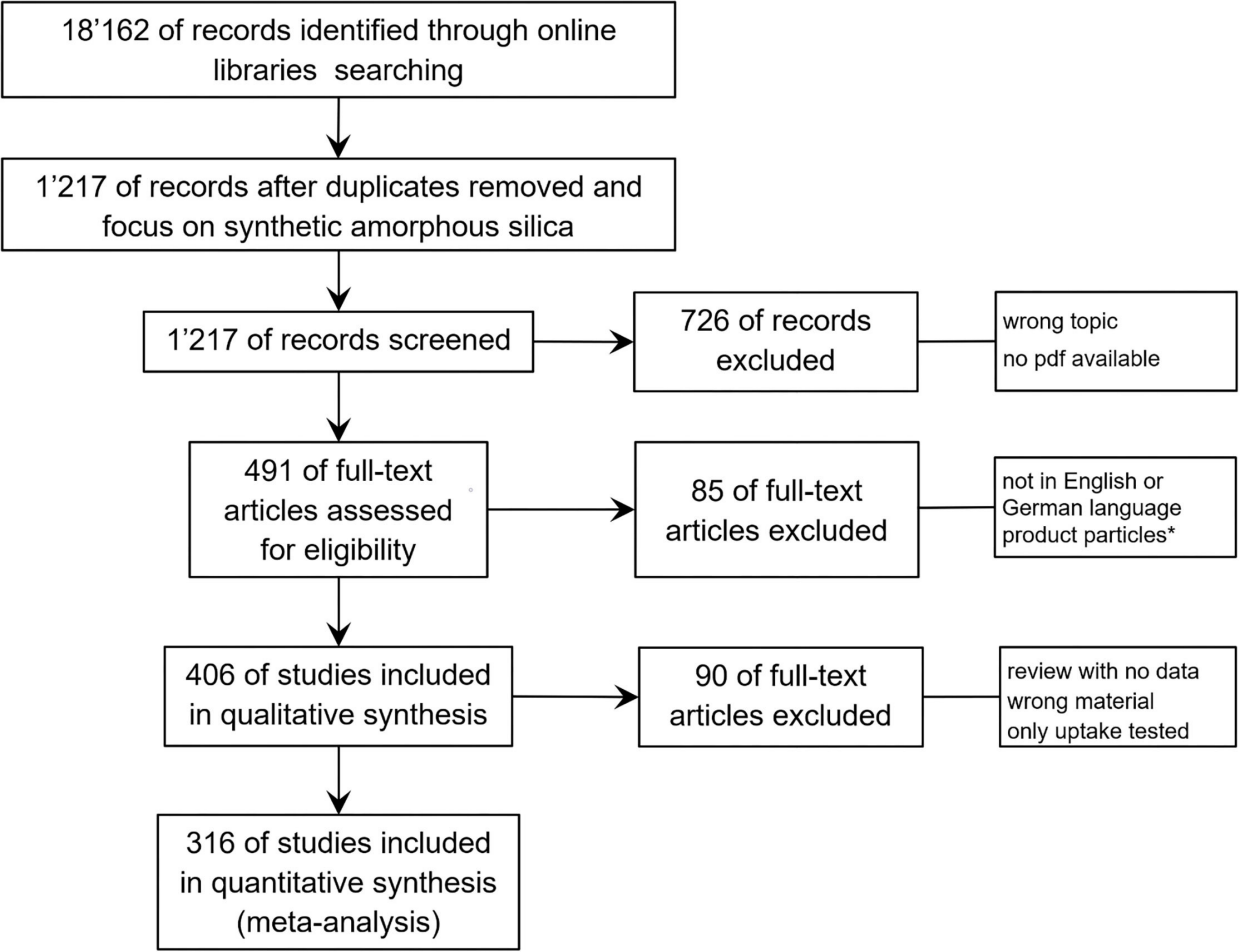
PRISMA flow diagram demonstrating the process of systematic selection of relevant publications for synthetic amorphous silica (SAS) safety studies. Starting with 18,162 citations for the period 2013–2018 on nanotoxicology, 1,217 have been selected with the keyword “synthetic amorphous silica.” Evaluating these studies in more detail, the final database contains 316 publications that have been assessed for all study details. *Studies with particle mixtures from commercially available products tested, such as paints, have been excluded.

The references that we found have been screened in the first round roughly for their toxicological content. Many publications use buzzwords, such as “toxic” or “toxicity” in the Introduction or Discussion section, without performing any toxicological experiment in the core test. These publications have been excluded by a quick pass through the literature. For this systematic review, all remaining studies are searched for “synthetic amorphous silica (SAS)” or “silica, excluding crystalline silica” again, and the resulting publications have been included in the evaluation of their eligibility. Further exclusion criteria in the next steps were as follows: (i) research not involving animals or cells or tissues and the material “amorphous silica” was only mentioned but not investigated; (ii) the study was not published in the English or the German language, and only particles from commercially available products, such as paints, have been analyzed; (iii) for the type of publication (review, conference abstract, editorial, etc.), the wrong material was used (crystalline silica, mixtures of silica with other oxides or polymers, etc.) or only uptake into tissues or cells were tested. The PRISMA flow diagram of the literature search and selection process is depicted in Figure 1.

### Quality Assessment of Studies

In the data collection, all publications from 2013 onward are re-evaluated regarding the quality of study details. Various suggestions exist on how the quality of studies could be assessed (14, 29–31). Detailed and quantitative evaluation is made possible by the criteria catalog of the European GUIDE nanoproject (29). This tabular record of study data was expanded for automatic use, and this was completed for all publications. For detailed information on which criteria are important and have to be given in the published studies, please refer to Table 5 (*in vivo* studies), Table 6 (*in vitro* studies), and Table 9 (material characterization) in the cited reference (29). Evaluation factors for characterization of the materials used (S-factor) and the toxicological study (*in vitro, in vivo*, or ecotox = K-factor) are offset against each other and resulted in four different values for quality (Q-score): “0” means no reliable study, “0.5” means low reliability, “0.8” means good reliable study, and “1” means very reliable study.

### Data Extraction and Analysis

Besides the bibliographic information of each study, relevant data from the publications were extracted and put in the data collection in four different groups. The first section regards the material properties given by the authors, including source, size of primary particles, specific surface area, and all additional information available. The second section contains information about the investigated biological model, such as species, strain, source of animals or cell type, and source for cell and tissue cultures. All details of housing, medium, exposure pathway, treatment design and duration, repeats, etc., are collected for the second section. The third section reflects the used doses or concentrations for testing and additional information about repeated-dose experiments, recovery and observation time, overload scenarios, or more details about the amount of material used for experiments. Finally, the fourth section is related to the biological endpoints investigated and which methods were used. The table contains for each investigated endpoint the no-observed-adverse-effect-level (NOAEL) for *in vivo* studies or the no-observed-effect-concentration (NOEC or EC_0_) for *in vitro* studies. Not all studies allow for direct determination of the NOAEL/EC_0_ from the data because the concentration range investigated was not chosen properly. Therefore, in such a case, either the lowest-observed-adverse-effect-level (LOAEL) or the lowest-observed-effect-concentration (LOEC) is given if even the lowest dose used in the experiment still induced an effect, or the upper-no-observed-effect-level (UNOEL) or upper-no-observed-effect-concentration (UNOEC) if even the highest dose used did not induce an effect *in vivo* or *in vitro*, respectively.

### Possible Subjective Perception

This study on synthetic amorphous silica has been carried out by the author of this publication in person. The detailed pre-selection process of more than 1,000 publications on synthetic amorphous silica safety and evaluation detail of 316 studies took several months. The extraction of data and assessment of literature quality have been conducted in all conscience, but of course, there are personal limits to attention, and the author is aware of his subjectivity. Thus, it may have happened that several publications have not been considered for a certain issue or the results have been misinterpreted and differ from the opinion of the authors of the studies. As a result, the pictures drawn and the data presented are, to some extent, a personal view of the actual situation of the published data on amorphous silica nanotoxicological issues between 2013 and 2018.

## Results

### Study Characteristics

Searching the online libraries for nanotoxicological studies for the period 2013 to 2018 resulted in 18,162 references, which have been further selected following the PRISMA flow diagram shown in Figure 1. The number of publications was reduced by focusing on the material (SAS, synthetic amorphous silica), and exclusion of all studies showing no toxicological data or in which one of the other exclusion criteria were applied (Figure 1). This resulted in 316 publications providing 973 data sets on different SAS modifications studied in different cell types or animal species and presenting data for NOAEL or EC0 for more than 85 biological endpoints (key events or KEs). Most of the KEs are described only once or <5 times, and for 32 KEs more than 20 datasets exist.

At this point, it is worth noting that the above-mentioned SAS modifications refer to the large number of chemical processes used for the synthesis of SAS in all the studies. The processes do not fulfill, in most cases, the criteria of market production. The most common manufacturing processes are pyrogenic (fumed) silica, precipitated silica or silica gel for powders, and colloidal silica for dispersions. Depending on the process, the final material reaches different states of aggregation (32). Pyrogenic (fumed) silica, precipitated silica, and silica gel are nanostructured materials. The particle size is characterized by different levels of structures, namely, internal structures, aggregates, and agglomerates. Internal structures (also referred to as primary particles or constituent particles) could be visually recognized by TEM because of their curvature inside the aggregate skeleton, but they cannot be isolated. There are no phase boundaries inside an aggregate (32). The typical size of internal structures ranges from ~3 to 50 nm (d50, number-based, TEM). Aggregates represent the smallest dispersible unit in synthetic amorphous silica and are formed during the production process by the fusion of primary structures by covalent bonds. Aggregates (33) usually have a particle size above 100 nm, although some fractions below 100 nm may occur. Agglomerates are formed out of aggregates by weaker forces, e.g., the Van der Waals force. Synthetic amorphous silica is usually placed on the market in the form of agglomerates. The typical size of agglomerates is >1 μm.

Several studies have been performed with isolated individual particles, which can be achieved by surface treatment or different dispersion methods. For evaluation of size-dependent effects, the authors' information on primary particle size was used without considering aggregation or agglomeration. Primary particle size is usually analyzed visually in the studies by transmission electron microscopy. This information is important for the comparison of the shown data with real market products, such as E 551 (food-grade SAS).

For all publications in the data collection, a rigorous quality check was conducted using the quality criteria checklist established by the EU project GUIDEnano. Only 3.4% of the studies reached the highest classification of “1” (very reliable) and 24% reached the level “0.8” for good reliability. Conversely, however, this also means that more than 70% of the studies do not provide reliable data. A comparison to quality classification regarding the literature published before 2013 (9, 10) resulted in no increase in the reliability of data regarding the toxicological content of the more recent studies evaluated in this systematic review.

### General Results

Although the overall number of datasets is high, the range of variation in the different material modifications (Table 1) and the tested biological models (Table 2) is very high as well, which considerably worsens the comparability of the results. Several studies used core-shell materials for which the shell was made from amorphous silica most often following the water-glass method. These silica-coated materials (core-shell particles, Table 1, last column) were taken into consideration as well, as they demonstrated the “detoxification” effect of different materials using the silica shell. Eleven different materials, such as gold, zinc oxide, and iron oxide, coated with amorphous silica were used in 21 studies mostly to reduce the toxicity of the core material (34–42). Also of significance is the fact that only 14 datasets resulted from experiments with food-grade silica, which is the most relevant modification for human toxicity testing, and four of these datasets were questionable because no source or product number was given; whereas 140 datasets were produced with technically specified SAS made for a huge variety of products, such as paints, surface coatings, rubber, and plastics, and most of the studies used unspecified SAS or self-made silica particles. In many cases, material properties were not analyzed sufficiently, such as surface charge and specific surface area, and were mentioned by the authors in only 52 and 27% of the studies, respectively.

**Table 1:** Modifications of amorphous silica used in the 316 selected studies.

	**Amorphous silica modification**					
**Type of material**	**Technical food-grade (SAS)**	**Technical non-food-grade (SAS)**	**Technical—unspecified**	**Self-made (unknown process)**	**Self-made (Stöber process)**	**Core-shell particles (shell made from amorphous silica)**
# of datasets	14	140	448	212	123	36

**Table 2:** Biological models (animals or cell and tissue cultures) used in the 316 selected studies.

	**Animal models**									**Cell models (170 different)**	
**Species**	**Cnidaria**	**Sea urchin**	**Nematode**	**Fish**	**Fly**	**Chicken**	**Mouse**	**Rat**	**Rabbit**	**Human**	**Animal**
# of datasets	1	1	3	25	6	1	125	79	3	447	282

In addition, the range of variation in the experiments is further increased by the fact that very different concentration units for the treatment of cells or animals were given in the experiments (Table 3). Moreover, some of the units used did not make any sense if in combination with nanostructured materials or nanoparticles, as it is difficult or impossible to repeat experiments with the given concentration of nM or μM for particulate materials.

**Table 3:** Dose and concentration units applied in the experiments of the 316 studies on amorphous silica toxicity.

**Concentration units (*in vitro* studies)**	**Dose units (*in vivo* studies)**
#/cell	μg or mg/animal
#/ml	μg or mg/kg
μg/cm^2^	mg/m^3^
μg/ml	μg/area skin
μg or mg/plate or well	μg/ear
μl	μg/eye
nM or μM	#/animal
ppm	μg/embryo

The data collection additionally recorded typical material characteristics, such as the size of the primary particles or the shape of the materials. To discriminate between size-dependent effects, the data were classified into six different size groups regarding primary particle size given in the studies (Table 4). As mentioned above, during the synthesis process, frequent aggregation and agglomeration took place, which was described in 332 datasets (roughly 30% of all the datasets). Here, the particle sizes are between 100 nm and often far above 1 μm. Moreover, some materials had a very special shape (rods, nanowires, spindles, rattles, or irregular aspects were used in 28 datasets), but most of the experiments were carried out with aggregates or agglomerates from spherical primary particles (Table 4).

**Table 4:** Distribution of the datasets over the size classes and different material forms.

	**Distribution of datasets over six size groups**					
**Size group (nm)**	**0–10**	**11–20**	**21–50**	**51–100**	**101–500**	**>500**
# of datasets	70	250	269	209	108	67
	**Occurrence of different primary particle shapes** ^**#**^					
**Shape**	Spherical*	**Rods**	**Nanowires**	**Spindles**	**Irregular**	**Rattles**
# of datasets	908	8	1	2	14	3

*^#^For the 37 missing datasets, no information on shape was available [no transmission electron microscopy (TEM) pictures are shown or no information is given by the authors of the studies]*.

^*^Synthetic amorphous silica (SAS) produced for the market belongs to the spherical form.

Most of the studies presented data on specific biological endpoints or key events. These played a role in different pathways of toxicity (43) or adverse outcome pathways (44). Table 5 depicts the most important pathways of toxicity (PoT) that have been addressed in the studies and the number of datasets existing for each of the PoT. Induction of a PoT does not imply that the material is highly toxic, as no concentration or dose-relationship is included in this table, and mostly only high concentrations have induced the respective key event.

**Table 5:** Involved pathways of toxicity in the studies on amorphous silica as mentioned by the authors.

**PoT**	**Cell viability**	**Apoptosis**	**Membrano-lysis**	**Oxidative stress**	**Stress kinases**	**Immune response**	**Inflammation**	**Gene expression**	**DNA damage**	**Tissue protection**	**No-effect study**
# of datasets	91	27	3	88	11	8	128	11	17	8	456
Quality^*^ rel./not rel.	22/69	7/20	0/3	22/66	5/6	3/5	42/86	3/8	9/8	3/5	139/317

^*^ Quality as evaluated; rel., reliable (Q-score 0.8 or 1); not rel., not reliable (Q-score 0 or 0.5).

### *In vivo* Results

Most of the *in vivo* studies on amorphous silica were carried out with mice (45) or rats (37), and other organisms (Table 2) played only a minor role. The main exposure pathways chosen by the authors of the studies were intraperitoneal (IP) or intravenous (IV) injection, intratracheal instillation or aspiration, inhalation, and ingestion. For a better comparison of the results only those studies have been selected for the following detailed discussion which have applied “μg/kg bodyweight” in injection or instillation experiments and “mg/m^3^” for inhalation studies.

#### IP Injection

Only seven studies used this exposure pathway, four of them injected amorphous silica, and three studies used other materials with a silica coating. The main effects described were oxidative stress and inflammatory responses, but as all the studies got a quality score of “0,” they were not further considered.

#### IV Injection

Direct exposure by IV injection of amorphous silica particles was carried out in 23 studies. Half of the datasets showed no effect even at very high doses. There was only one study that discussed DNA damage based on observed p53 activation, but this effect was described at 50 mg/kg only in rats and could not be confirmed by comet assay (insignificant) as discussed by the authors (46). For this exposure pathway, the most often observed effect was liver injury (18 of 63 datasets), which is hardly surprising after the injection of doses in nearly all cases above 10–250 mg/kg. Only one study with a low-reliability score (“0”) showed impairment of survival of mice at concentrations of 17.5 mg/kg and above with 10-nm amorphous silica particles (47). Taken together, all the results of IV injection of amorphous silica particles in rats and mice only induced slight effects at very high doses.

#### Intratracheal Instillation

If administered by instillation or aspiration, the dose rates were very high (48). Moreover, in most of the studies, relatively high doses were applied. Within the five repeated exposure studies in mice (3 to 14 repeats), 1 to 25 mg/kg bw have been instilled which results in overload of the lungs except in only one study. In the studies carried out with rats, 75–125 mg/kg bw were instilled in single and repeated-dose experiments, which all ended up with overload doses. Because of these facts, high doses and high dose rates, and inflammatory effects, such as immune cell migration and cytokine production, were observed in most studies, which is the normal tissue response under these conditions. Nevertheless, two-thirds of the studies were not reliable based on the evaluated quality criteria, and many of them went beyond overload concentrations for rats (≥2.5 mg per lung) and mice (≥0.5 mg per lung) (45, 49–62).

#### Inhalation

The number of inhalation studies was generally low, and in this period, only six studies on amorphous silica or silica-coated materials were found. Only two studies had a low-reliability score and were not considered here. The other four studies, three on rats and one on mice showed no effects on the lungs of the treated animals (63–66) except for the study of Leppanen et al. (66) who used 10–40 nm rod-shaped silica-coated titanium dioxide particles at overload dose (exposure treatment: 30 mg/m^3^, 4 days a week, 4 weeks, calculated deposited dose 575 μg/lung in mice). One study with high reliability investigated five different surface modifications of SAS following the OECD guideline 412/403 (65). For the unchanged pristine material (15 nm amorphous silica) a NOAEL of 2.5 mg/m^3^ has been observed, whereas all coated silica materials (acrylate, PEG, phosphate, NH_2_) had no effect even at the highest used dose and the NOAEL was stated to be higher than 50 mg/m^3^. The only effect described for the coated materials was the acrylate modified amorphous silica induced some increase in weight of the spleen and accumulation of nanoparticles and thrombocytes at a NOAEL of 0.5 mg/m^3^. However, this effect was fully reversible after the treatment phase.

#### Ingestion

The data collection contains 14 studies that exposed animals *via* the gastrointestinal tract resulting in 35 datasets. Completely unexpectedly, none of the studies was performed with food-grade silica, and only five followed the OECD guidelines.

Test Guideline 408: Repeated Dose 90-day Oral Toxicity Study in Rodents (67, 68).
Test Guideline 414: Prenatal Developmental Toxicity Study (69).
Test Guideline 416: Two-Generation Reproduction Toxicity Study (70).
Test Guideline 420: Acute Oral Toxicity: Fixed Dose Procedure (67).
Test Guideline 474: Mammalian Erythrocyte Micronucleus Test (71).

Only four of the 14 studies fulfilled the criteria for quality scoring of good studies (67, 69, 72, 73), and a fifth study used a well-characterized material from the JRC repository but without any own detailed analysis especially for contaminants (70). The four studies were conducted with rats except for one with very small particles (between 12 and 26 nm). All of these were “no-effect studies,” although some exposed the animals over 90 days to relatively high doses (up to 1,500 mg/kg bw/day). In the high-exposure scenario chosen by Liang et al. (72), only some histopathological observations on the liver and lungs were conducted, but the control animals had slightly higher liver degeneration compared to the treated groups. Moreover, in the same study, two different sizes of silica particles were tested, one with 26-nm primary particle size and the bigger one with >1 μm; both have the same weak effect on the lungs, but there were no effects on survival and body weight, and there were no hematological changes.

The ingestion pathway is of special interest as amorphous silica is a registered food ingredient. For this reason, the studies with low-quality scores were also included for a detailed analysis. The low-quality studies did not change the overall picture of the low toxicity of amorphous silica. One study was scored “0” for quality because of missing material characterization showing DNA damage in peripheral blood lymphocytes after a single dose of 3, 7, 10, and 13 mg/kg bw silica (74), but none of the other studies could find a similar effect. In another study on mice with only one dose tested (750 mg/kg bw/day for 14 days), the authors observed some effects on cytokines and oxidative responses with colloidal silica particles of two different sizes (20 and 90 nm) and two different coatings (citrate and L-arginine) (75). In the case of citrate-coated SAS, Il-12p70 was reduced to some extent and arginine-coated particles induced intracellular reactive oxygen species (ROS) production slightly. All shown effects were very weak or statistically insignificant. The authors stressed the point that their experiments demonstrated immunosuppression, which was not confirmed by the results as the materials were not characterized very carefully, and no contaminants or endotoxins were analyzed.

### *In vitro* Results

The number of *in vitro* studies was much larger than that of *in vivo* studies. A total of 729 datasets represented a good basis for reliable statements. Nevertheless, the overall number of studies with comparable experimental study designs was still low; most studies were not conducted using standardized protocols. Most of the experiments were carried out under submersed conditions. More complex exposure methods, such as the air-liquid interface method, to compare inhalation conditions were also established, and as of 2018, 15 datasets for amorphous silica were available for this exposure method (76). Moreover, the incubation conditions also played a role. Cells in their normal environment “see” a mixture of biological molecules, including proteins and peptides, thus the data presented here were chosen from experiments carried out with full medium-containing serum. A total of 180 datasets showed results with coated or functionalized particles. The coatings/functionalization can be mainly divided into 4 classes: fluorescence dye molecules, polymers, carboxylation, and amination. Although the match in the design of the experiments was relatively low, often, different assays were used and quite different concentration units were given (Table 3), and there remained sufficiently large group numbers for some of the endpoints investigated to illustrate the results graphically. There were several important experimental variables, such as dose metric and treatment period. For this reason, certain limitations were placed on the comparative analysis. To increase the comparability, only datasets that performed 24-h incubation were chosen for the following evaluation. For the applied dose, only data from studies that presented concentration as μg/ml or if the concentration could be recalculated to this unit by the information provided by the authors were included. Regarding the concentration of nanomaterials, a certain degree of uncertainty remains, since, in a large number of publications, the exact amount of nanomaterials applied cannot be re-calculated because of a lack of information on the number of cells or the volume of the medium above the cells.

**Figure 2:**
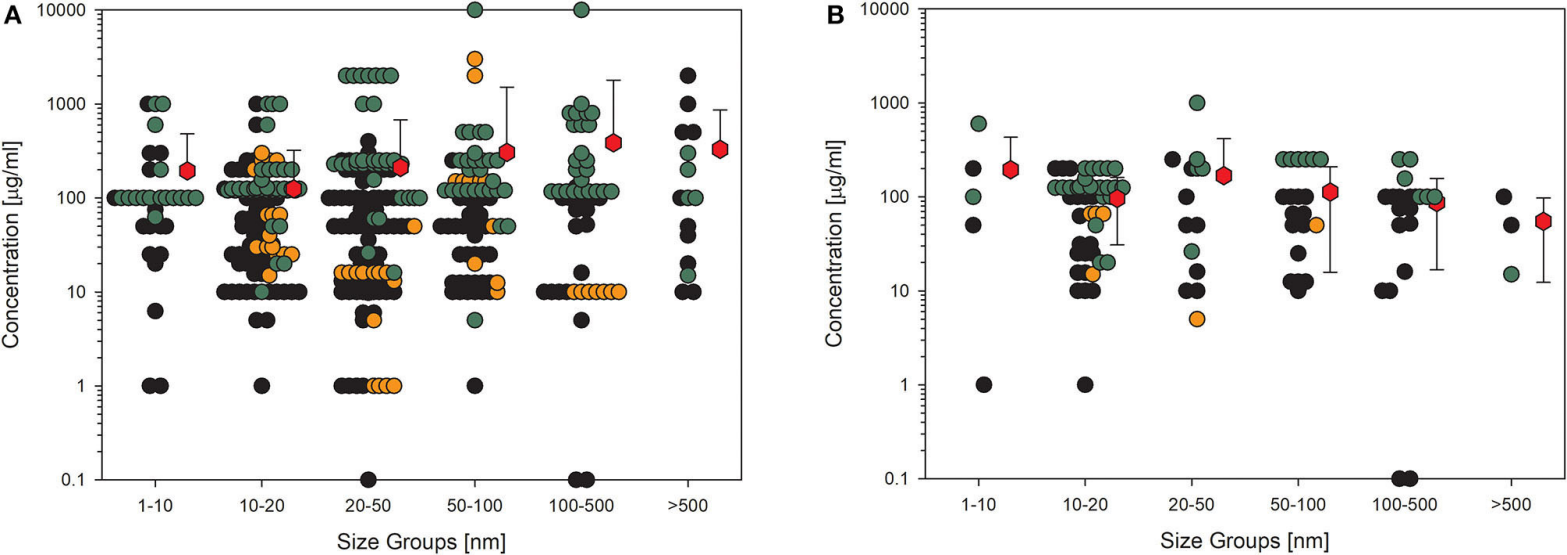
Results of the evaluation of *in vitro* studies on induction of cell death in cells treated with amorphous silica. Data have been collected in six size groups for primary particle size as indicated on the abscissa. **(A)** All available datasets; **(B)** datasets from studies with high-quality scores of 0.8 or 1. Shown are the concentrations given in the publications for the no-observed-effect concentration (EC_0_): black circles (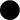) or the lowest-observed-effect concentration (LOEC; if no EC_0_ was available): orange circles (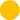) the upper-no-observed-effect concentration (UNOEC; if the highest tested concentration could not induce the investigated effect): green circles (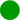). The red hexagons (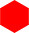) indicate the mean of all data for one size group ± SD. Data points taken from the references are given in the Supplementary Material.

Biological endpoints (key events) with the highest number of datasets were chosen for further presentation in detail. The four key events were cytotoxicity, most often measured by MTT assay, represented by 403 datasets; membrane integrity, analyzed by LDH measurement or trypan blue staining, with 231 datasets; detection of ROS, usually tested by dichloro-dihydrofluorescein diacetate (DCFH-DA) assay and represented by 167 datasets; cytokine production, most often tested by ELISA and represented by 116 datasets. To assess the influence of quality evaluation on the appearance of the data, the next figures show the data points for (A) all available datasets and in the other figure those for (B) the high-quality studies only. Moreover, the data are distributed in six size groups (compare Table 4) to be able to recognize a possible size-dependent effect regarding the primary particle size of the investigated material. The following figures show data points in three different colors. This is to represent the three different categories of concentrations, the NOEC or EC_0_ is shown with black dots, the LOEC is shown in orange dots, and the green dots represent the values for the UNOEC. This also explains the appearance of the orange dots often at the lower concentration range and the green dots frequently at the higher concentration range. Figure 2 shows the first example of the possible cytotoxic effect of amorphous silica on cells.

The data shown in Figure 2 impressively show how high the variance is in the different studies. The values within one size group scatter over more than five orders of magnitude, which demonstrates the missing use of standardized protocols. The same has been shown for ecotoxicological studies in which the values are distributed over up to six orders of magnitude (77). Reducing the data points to only the high-quality studies (Figure 2B) reduces the distribution for all size groups but has nearly no influence on the size of the mean values, which is, in all cases, larger than 100 μg/ml except for the largest size group and high-quality studies. The mean is relatively the same for all the six size groups, indicating that there appears to be no size dependence for this endpoint. Also striking are the recognizable outliers at very low concentrations, which can be explained as follows: the EC_0_ of 0.1 μg/ml in the 20–50 nm group is due to amorphous silica coated with amino groups, which significantly increases toxicity. The two data points in the 100–500 nm group are due to a self-made material with the Stöber-method (78), and the reduction in viability at concentrations lower than 10 μg/ml is very small.

**Figure 3:**
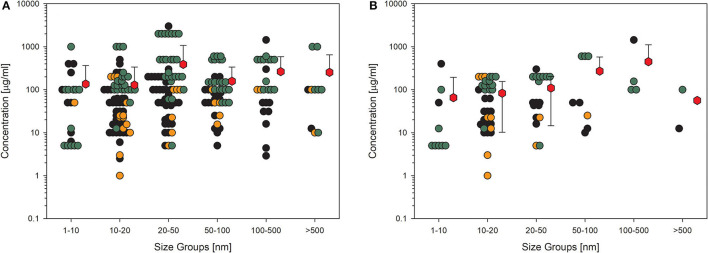
Results of the evaluation of *in vitro* studies on disruption of the membrane integrity of cells treated with amorphous silica. Data have been collected in six size groups for primary particle size as indicated on the abscissa. **(A)** All available datasets; **(B)** datasets from studies with high-quality scores of 0.8 or 1. Details are as those shown in Figure 2. Data points taken from the references are given in the Supplementary Material.

A similar picture results from the data on membrane integrity of the treated cells (Figures 3A,B). The values here also lie in the same range, although the overall number of data points is lower. This is more important if only the high-quality studies are considered (Figure 3B), which reduces the number of data points dramatically, and for some size groups, no standard deviation can be calculated because of the low number of values. Nevertheless, nearly all the mean values are above 100 μg/ml.

**Figure 4:**
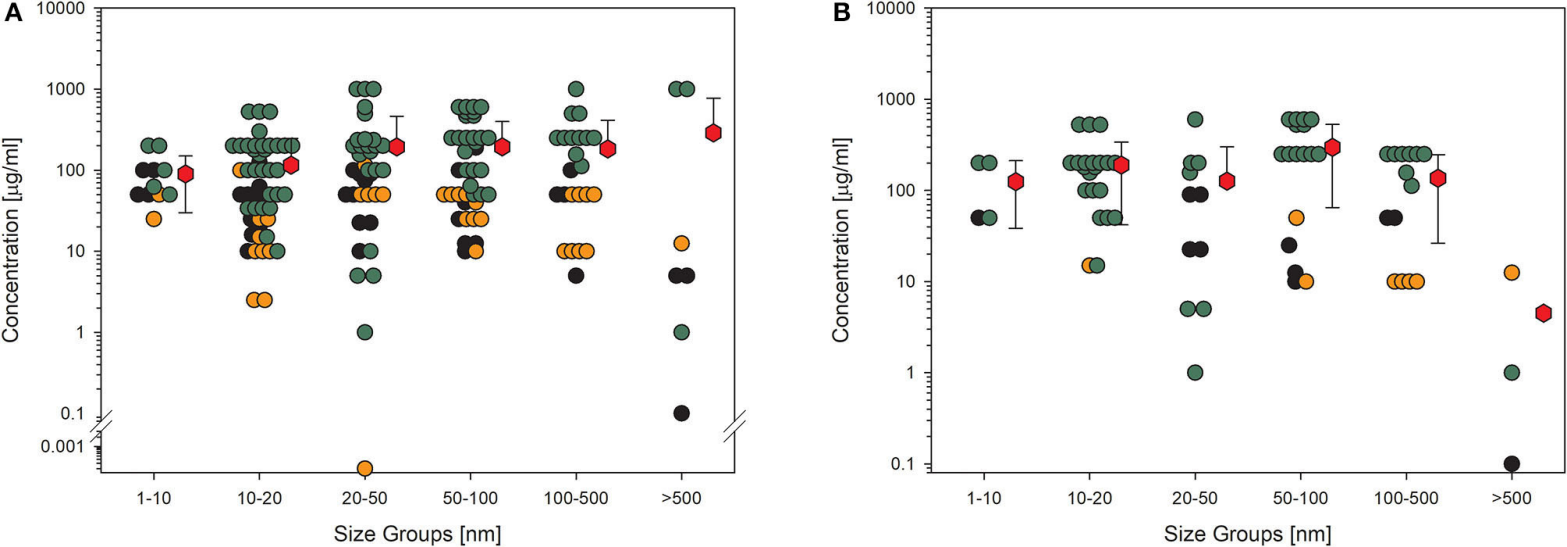
Results of the evaluation of *in vitro* studies on induction of ROS production in cells treated with amorphous silica. Data have been collected in six size groups for primary particle size as indicated on the abscissa. **(A)** All available datasets; **(B)** datasets from studies with high-quality scores of 0.8 or 1. Details are as those shown in Figure 2. Data points taken from the references are given in the Supplementary Material.

A common endpoint related to nanomaterial exposure is the formation of ROS and oxidative stress (79). This is possibly the reason why many studies included this endpoint in their experiments (Figures 4A,B). The entire dataset for this KE paints an identical picture compared to the acute toxicity testing. Mean values are above 100 μg/ml, except for the largest particle group and high-quality studies, and for this case, too few values are available (Figure 4B). Again, some outliers are obvious, with one concentration being extremely low in the group of 20- to 50-nm-sized particles (Figure 4A). This study reached only a low-quality evaluation and was carried out with 35 nm amorphous silica particles bought from a company (80). Contaminants were not analyzed, and the dilution steps for this extremely low concentration applied were not explained in the study description. This is the only study demonstrating an effect at such low concentrations of nanoparticles. Compared to the huge amount of green data points, which means that the concentrations are not able to induce the formation of various oxygen species, this outcome may be questionable.

**Figure 5:**
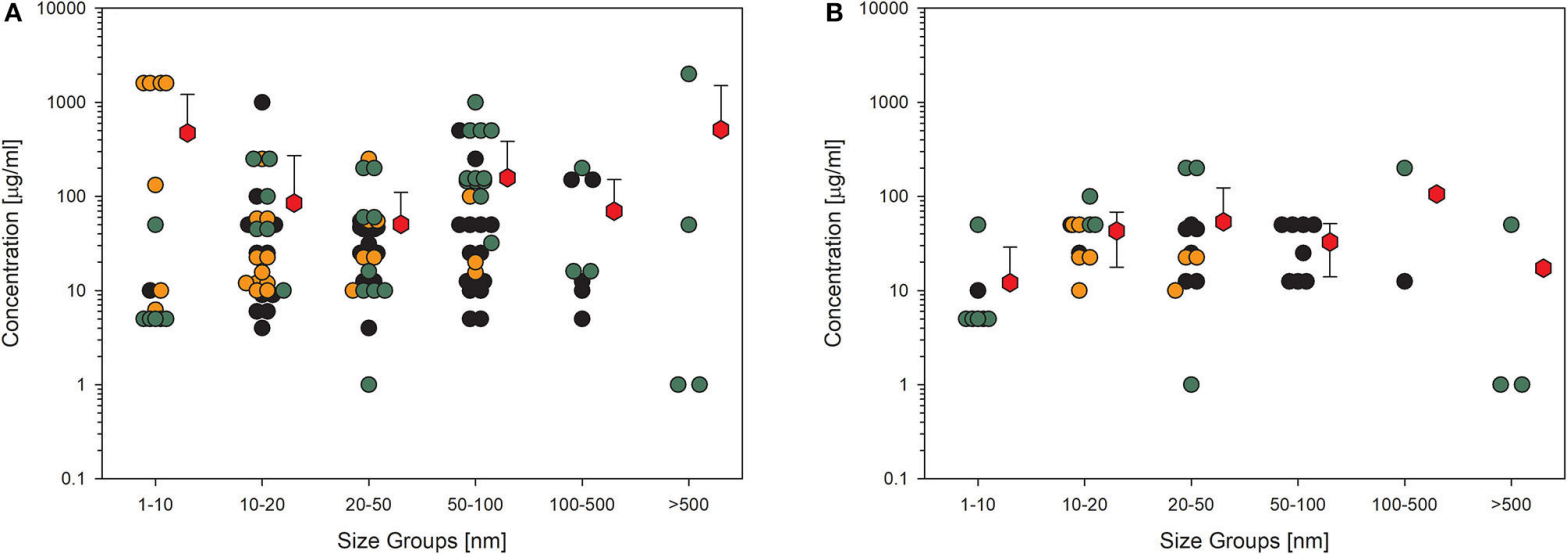
Results of the evaluation of *in vitro* studies on cytokine production in cells treated with amorphous silica. Data have been collected in six size groups for primary particle size as indicated on the abscissa. **(A)** All available datasets; **(B)** datasets from studies with high-quality scores of 0.8 or 1. Details are as those shown in Figure 2. Data points taken from the references are given in the Supplementary Material.

The last example shows data for another biological endpoint, which is representative of classical cell response, production, and release of cytokines. Although this KE is very important for the assessment of further adverse outcome pathways, such as fibrosis, it is not as much investigated as the other shown endpoints. This implies higher uncertainty in the statistical significance even if the mean values are all above 50 μg/ml (Figure 5A). In this case, it is also noticeable when the studies with low quality are omitted (Figure 5B), then, the number of remaining data points is so low that a reliable statement is no longer possible. Then, the number of remaining data points is so low that a reliable statement is no longer possible.

**Figure 6:**
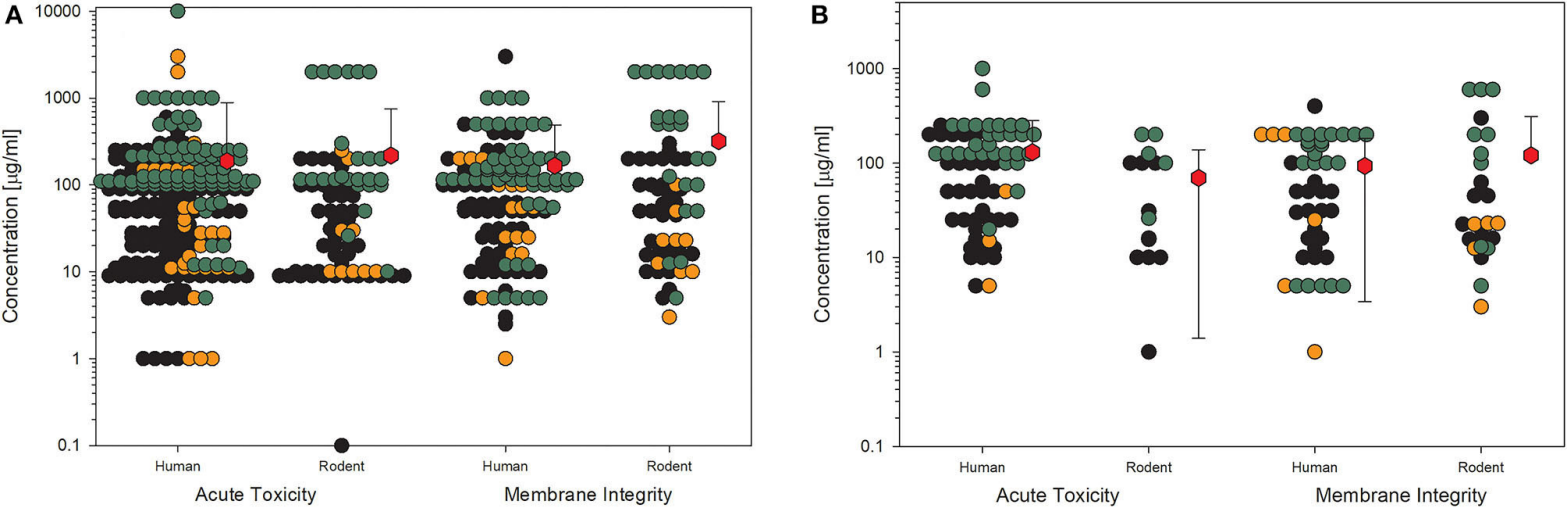
Comparison of human and rodent cell lines in terms of their sensitivity to amorphous silica treatment. Datasets on the key events “acute toxicity” and “membrane integrity” are shown. For this comparison, only data for particles with primary sizes below 100 nm were included. Mean values are calculated from each dot cloud (red hexagons). **(A)** All available datasets; **(B)** datasets from studies with high-quality scores of 0.8 or 1. Details are as those shown in Figure 2.

**Figure 7:**
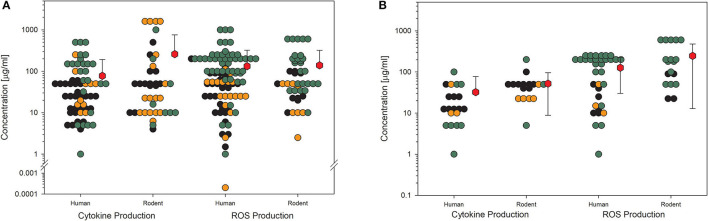
Comparison of human and rodent cell lines in terms of their sensitivity to amorphous silica treatment. Datasets on the key events “cytokine production” and “ROS production” are shown. For this comparison, only data for particles with primary sizes below 100 nm were included. Mean values are calculated from each dot cloud (red hexagons). **(A)** All available datasets; **(B)** datasets from studies with high-quality scores of 0.8 or 1. Details are as those shown in Figure 2.

For toxicological studies, it is often the question of which biological model should be used. So far, the gold standard seems to be animal testing. However, several studies have shown the limitations of animal studies, which sometimes have higher uncertainty than cell culture experiments (81). Moreover, the same group around Thomas Hartung has shown in an internationally acclaimed study that the readout of big data from existing toxicological data is outperforming animal testing (82). Furthermore, cell culture systems increase in complexity, reflecting more and more the situation in the whole organ and thereby replacing animal studies (83). Going through the collection of data on nanomaterial toxicology used for this study, the number of *in vitro* models is tremendously high. The authors of the studies often look for similarities in tissues of the exposure pathway of interest, e.g., lung epithelial or gastrointestinal tract cells. Moreover, to compare the results between animals and cell cultures, the respective cells should come from the same species, and to compare the results to humans, the cell culture models should reflect the tissues of both species, animals, and humans (2). Finally, the comparison between cellular responses to nanomaterials of animal cells and human cells might be of interest to demonstrate if there is a difference in sensitivity or not. The same four KEs, which have been shown above, have been analyzed with the existing data on amorphous silica studies for possible differences in the biological response in rodent cell cultures compared to human cell cultures (Figures 6, 7). The data from the literature between 2013 and 2018 justifies the statement that there is no difference in sensitivity to amorphous silica between human and rodent cell lines. All four shown KEs do not show any differences between both types independent of the quality of the studies. The mean values are nearly identical not only for the different species but also for all the four KEs. The lowest mean value can be seen for cytokine production in human cells for high-quality studies with 32 μg/ml (Figure 7B). All the other mean values are above 50 μg/ml, mostly far above 100 μg/ml.

## Discussion

This systematic review focused on synthetic amorphous silica (SAS), a material that has been produced for more than 80 years, has been investigated in hundreds of studies, and is used in cosmetics, pharmaceuticals, as a food additive, and special modifications of many other products. Important is the fact that all publications selected from the available libraries have been evaluated regarding the mentioned “primary particle sizes” of the used amorphous silica materials. Dry powder materials produced for the market exist as aggregates and agglomerates with sizes ranging from several hundreds of nm to above 1 μm. Aggregates are individual indivisible units without well-defined physical boundaries between the primary particles (32). Primary particles usually do not exist in the products on the market but are often produced specifically for the studies described in this review. To better isolate the particles, they are often sonicated with different forms of energy to get the particles in suspension. Moreover, suspensions of primary particles are often stabilized by additives or applied surface charges, and these variables make studies even more diverse. Thus, the comparability of the published data is severely limited and must be interpreted very cautiously. Nevertheless, this overview of the effects of amorphous silica and SAS *in vivo* and *in vitro* attempts to provide a good insight into current nanotoxicological studies as has been shown for other materials as well (9, 10, 84).

A key point of this literature review is to assess the quality of studies regarding their nanotoxicological potential. It has been assumed that over time, with most national- and international-funded projects, the quality of toxicological publications will increase. During the last two decades, unfortunately, this has not been the case. The evaluation of studies from the first decade of the century showed that only 30% of the studies provided reliable data (9, 10, 13), and this low reliability was also evident in the evaluation of studies from the subsequent decade, as shown in this and other evaluations (29). In order not to be too critical, it should also be mentioned that a significantly higher level of quality has been achieved in ecotoxicological studies with algae (47%) and fish (63%) (29).

Selective assessment of the four most often investigated biological endpoints *in vitro* (85) and most important exposure scenarios in animal studies leave no doubt that amorphous silica and, especially SAS, are non-toxic. It is noticeable that there appear from time-to-time publications with special conditions and materials that observe serious effects, but these are usually not representative of the materials on the market. For example, all animal studies on gastrointestinal exposure were conducted with non-food amorphous silica in this period, but in these publications, it is then discussed that “SAS” as a food additive could have negative consequences on consumers, which is a wrong conclusion. Avoiding this problem is only possible if material characterization will be intensified and the relationship to real-life scenarios will not be forgotten in the study design of toxicological experiments.

For various nanomaterials, it could be observed that the strength of a biological effect depends on the size of primary particles or, better, on the increased specific surface area of smaller particles compared to their bigger counterparts. Twenty years ago, Oberdörster (86) published important data on the comparison of ultrafine and fine titanium dioxide demonstrating that effects after lung exposure are quantitatively size-dependent on the particles applied. Following the first principle in nanotoxicology, the transport principle (2), this might be due to better transport of smaller particles, as this could be clearly shown in studies on the human placenta (87) and the lungs (88, 89) or a combined effect of transport and faster solubility because of the larger surface area shown for another material than the one investigated in this study, silver nanoparticles (90). On the other hand, there are also publications demonstrating total independence from particle size (91, 92) or a direct relationship to the applied mass of the nanomaterial (93). This review and another publication in parallel demonstrated *in vitro* experiments with no size-dependent effects for amorphous silica (this study) or titanium dioxide (8). Moreover, most of the animal studies evaluated in this systematic review do not show a size-dependent effect either (data not shown), but the statistical significance of this statement is weak because of the low number of comparable studies and the low potential to induce biological effects of the amorphous silica material.

Certain parameters exist in the publications that have a severe influence on the interpretation of the results and the repeatability of the experiments. The most common flaw is still the missing information about the exact cell number or the exact experimental volume of each sample for *in vitro* experiments. The simple information “the cells have been treated with 20 μg nanomaterial per milliliter” is not enough to recalculate the exact amount of nanomaterial the cells have “seen” in this experiment. During the experiments, a certain Petri dish with a specific number of cells may be filled with 0.5, 1, or 2 ml of a nanomaterial suspension. This results in an overall amount of 10 μg in the first case, 20 μg in the second, and 40 μg in the third case. The difference in dose for the cells is 1:4! The same is true for the dose metric μg/cm^2^ if the cell number and surface area of the Petri dish are not given in parallel. There is a perpetual discussion among scientists about dose-metrics in nanotoxicology, and the most appropriate units have been suggested to be mass per volume (more traditional), mass per surface area or surface area per cell, and particle number per volume or cell (94–99). It does not matter which unit is chosen for an experiment; the most important criterion remains to be the traceability or repeatability of studies. As described above, the specification of mass per volume is not sufficient to unambiguously repeat an experiment, it is also necessary to know how much total volume was used in the treated sample and, of course, the treated cell number per sample. The same applies to the unit mass per surface. Here, the surface area of cells and the surface area of vessels must also be specified, since no repeatability is possible without this information. The unit particle surface per cell or cell number, on the other hand, is less suitable for normal laboratory use, since the falsification of the dose would be enormous here because of the different agglomeration and aggregation status of the nanoparticles. The same applies to particle number per cell number since here, the question also arises as to which particles are counted. Are they isolated primary particles or agglomerates/aggregates, and how are they isolated? For good traceability, therefore, mass per volume or surface area data are suitable for routine operation, but with the additional information outlined above, without which such data make no sense.

Another aspect is the so-called “landslide effect”. It has been demonstrated by Wittmaack (100, 101) that a concentration of 27 μg/ml of TiO_2_ applied to a cell culture on a 96-well-plate with a total amount of 8.4 or 25.2 μg/cm^2^ induces a total coverage of cells by the nanomaterial because of rapid sedimentation of particle agglomerates. This example makes clear that first, the dose unit μg/ml is susceptible to misinterpretations; second, concentrations above 30–50 μg/ml, referring to 20 or more μg/cm^2^, do not make sense for materials with a density higher than 1. The material will build up a layer of more than 50–100 nm in thickness, hindering nutrient and oxygen diffusion to the cell surface and leading to higher susceptibility to disturbing factors. Comparing the data in Figures 2–5, the majority of data points are produced with concentrations above this threshold, and, surprisingly, no higher toxicity was found. This might be another evidence of the low toxicity of amorphous silica.

The points presented above show the weaknesses of many studies, which may be the reason for the possible misinterpretation of their results. Detailed explanations should shortly demonstrate where reviewers of manuscripts must look closer in the future to increase the quality of published studies because “non-repeatability” reduces the reliability of experiments dramatically. Finally, in addition to the qualitative deficiencies that the studies exhibit, this review shows that there is low or no toxicity of synthetic amorphous silica and even of the self-made materials not produced for the market.

## Conclusion and Recommendations

### Conclusion

#### *In vivo* Data

- Systematic investigations on size dependency are missing. The overall number of studies that are comparable in the model, application route, dose, and test design is very limited.
- Data gaps exist in the published literature, especially for *in vivo* studies.
- To observe a trend for size dependency, the number of data points is too small to come to a significant conclusion.
- The only two studies out of six that performed inhalation experiments following OECD guidelines (63, 65) did not observe effects at 5 or 10 mg/m^3^, thus, the NOAEL can be higher.
- All effects observed *in vivo* after instillation or injection are induced at very high doses, mostly above 10 mg/kg.
- Oral application did not result in any effects. Not even after 90 days application of 2,000 mg/kg bw/day provoked any adverse response in rats.

#### *In vitro* Data

- As the number of data points increases, the quality of studies does not increase over time, as is hoped in various funding programs. Funded projects do not use the majority of the established protocols from former projects; thus, the quality of data is not better in the period 2013–2018 compared to the results for the period between 2000 and 2013 (data not shown).
- Size dependency is not apparent; all particles of all size groups have the same potency or, better, the same inertness.
- Outliers may be explained by sensitive models, wrong study design, or specific surface reactivity of self-made materials. The number of outliers is very few.
- No difference in sensitivity can be observed between human and animal cell lines.
- The mean concentrations of amorphous silica inducing biological effects in cells are around or above 100 μg/ml, which is far beyond reasonable concentrations.
- The systematic review of the literature on amorphous silica supports the hypothesis of the very low toxicity of amorphous silica to humans and animals. Especially considering the majority of “self-made” materials, which are not produced for the market under the restrictions of the law, it can be expected that relevant SAS produced for the market is much less harmful, and this leads to the overall conclusion that there is no reason for concern regarding the hazard of silica particles in the form of nanoparticles, larger particles, or agglomerates/aggregates.

### Recommendations

For future studies with animals, the study design and applied OECD guidelines should be carefully considered, as, without the use of harmonized protocols, toxicological studies on nanomaterials make no sense. The emphasis here is on the term “toxicological” because mechanistic studies should still be possible but should also be considered as such. To assess the hazardous effects of a given substance or material, dose-response relationships must be established for well-known biological endpoints. To classify a nanomaterial in a given scenario as the IARC classification for cancerogenicity, the key event is well-defined, and the experimental design is well-described (compare OECD Testing Guidelines). The study scenario is different from mechanistic studies. The objective of mechanistic studies is to look for the mode of action of a given substance or a material that often includes unknown endpoints and non-standardized protocols, which are newly developed or adopted. Exactly, this step of using non-standardized experimental protocols is a substantial difference from toxicological studies that should use SOPs, harmonized protocols, or OECD testing guidelines with well-defined biological endpoints in mind.

It has been often criticized that spurious data of high-dose experiments in single, not comparable studies are of little value (102).

It is of utmost importance to use only nanomaterials for toxicological studies that have been extensively characterized. To increase the quality of toxicological studies, the following prerequisites should be fulfilled for the underlying experiments:

1. a rigorous and adequate physicochemical characterization of the test materials is needed (compare mandatory and desirable properties at the DaNa4.0 website^4^);
2. adequate particle controls and appropriate positive controls for a specific biological endpoint should be included;
3. possible contaminants, such as endotoxins, should be analyzed;
4. interferences of the tested material with the assay should be excluded;
5. high dose experiments designed to produce toxicological effects, which are publishable (and sensational), should be avoided but
6. dose-response studies should cover the entire range, from no-effect concentration (EC_0_/NOAEL) to a concentration inducing biological response;
7. dosimetry should be meaningful and traceable;
8. sedimentation rate and cellular uptake should be considered;
9. improved sophisticated *in vitro* models (e.g., ALI) should be used to reflect more realistic conditions.

Evaluation of articles with specific regard to these points has sorted out 70–90% of the respective publications (9, 10). During the Quality Nano-project meeting in Heraklion, Greece in 2015, the first literature study on the content and quality of published studies between 2000 and 2013 was presented (9). The disappointing result of more than 75% of toxicological studies not meeting the quality standards was intensely discussed, and official representatives of the European Commission were hopeful that this will change with new projects. This has not become true, although several national and European projects established new SOPs and some published collections on the web (e.g., DaNa4.0^5^, EU-project PATROLS^6^). However, these SOPs will not be used in future projects as is obvious when comparing the outcomes of manifold published studies from more recent years. This systematic review of amorphous silica studies that were published during the period 2013 to 2018 shows clearly that only 3.4% of the studies reach the highest quality score of 1 and further 24% the level of 0.8. Thus, the recent literature fails to meet the expectations not only of the EU officers to show higher quality than earlier publications. For a future increase in the quality of toxicology-oriented studies, new funding programs at the national and international levels should insist that SOPs or OECD guidelines should be used in projects as a matter of principle besides own-developed protocols.

## Author Contributions

The author confirms being the sole contributor of this work and has approved it for publication.

## Funding

This study was supported from the Swiss Federal Office of Public Health (FOPH) and the German VCI (Association of the Chemical Industry) and the special review of amorphous silica materials and the open-access publication fees were funded by SAS for REACh.

## Conflict of Interest

SAS for REACh members assisted by critically reviewing the manuscript. Changes and suggestions were only accepted for the scientific content, but beyond that the funders had no influence on the design of the study, in the collection, analyses, or interpretation of data, or in the decision to publish the results. HK is Shareholder of his own company NanoCASE GmbH.

## Publisher's Note

All claims expressed in this article are solely those of the authors and do not necessarily represent those of their affiliated organizations, or those of the publisher, the editors and the reviewers. Any product that may be evaluated in this article, or claim that may be made by its manufacturer, is not guaranteed or endorsed by the publisher.

## Footnotes

^1^ https://www.nanosafetycluster.eu/outputs/nsc-compendium/

^2^ www.nanoobjects.info

^3^ https://nanopartikel.info/en/knowledge/literature-criteria-checklist/

^4^ https://nanopartikel.info/wp-content/uploads/2020/11/DaNa_criteria_checklist_2016tox_en.pdf

^5^ https://nanopartikel.info/en/knowledge/operating-instructions/

^6^ https://www.patrols-h2020.eu/publications/sops/index.php
