# Supplementary material for: A Systematic Review on the Hazard Assessment of Amorphous Silica Based on the Literature From 2013 to 2018
Source: Front Public Health. 2022 Jun 15;10:902893. doi: 10.3389/fpubh.2022.902893 (PMC9240267; doi:10.3389/fpubh.2022.902893)
Supplement: Supplementary file 1 [file Data_Sheet_1.PDF]

Supplementary Material

1 Search profile

Keyword search strategy used to find published nanotoxicological studies.

Field Delimiter	Search Field	Search Criterion	Search Term
	All Fields	Contains	nanotox*
OR	All Fields	Contains	fulleren* AND toxic*
OR	All Fields	Contains	carbo nanotube* AND toxic*
OR	All Fields	Contains	bucky ball* AND toxic
OR	All Fields	Contains	nanotube* AND toxic
OR	All Fields	Contains	nanoparticle* AND toxic*
OR	All Fields	Contains	nanomat* AND toxic*
OR	All Fields	Contains	nano* AND toxic
OR	Year	Contains	2013[1]

[1] for each year a separate search has been carried out. For yearly results see (1).
*: wildcard for the search words

Using this profile roughly 3'000 to 5'000 publications per year have been found. For the systematic review on amorphous silica all 18'162 records have been selected following the PRISMA flow diagram shown in Figure 1.

1. Krug HF. The uncertainty with nanosafety: Validity and reliability of published data. Colloids Surf B Biointerfaces. 2018;172:113-117.

2 Complete Reference List

Red: publications with quality score 0,8 (good reliability) and 1 (very good reliability)

[1]	S. S. Agnihothram, S. A. Vermudez, L. Mullis, T. A. Townsend, M. G. Manjanatha and M. P. Azevedo (2016): Silicon Dioxide Impedes Antiviral Response and Causes Genotoxic Insult During Calicivirus Replication. J Nanosci Nanotechnol, 16: 7720-7730. doi: 10.1166/jnn.2016.12828
[2]	M. Ahamed (2013): Silica nanoparticles-induced cytotoxicity, oxidative stress and apoptosis in cultured A431 and A549 cells. Hum Exp Toxicol, 32: 186-95. doi: 10.1177/0960327112459206
[3]	A. Ambrosone, M. R. Scotto di Vettimo, M. A. Malvindi, M. Roopin, O. Levy, V. Marchesano, P. P. Pompa, C. Tortiglione and A. Tino (2014): Impact of Amorphous SiO2

	Nanoparticles on a Living Organism: Morphological, Behavioral, and Molecular Biology Implications. Front Bioeng Biotechnol, 2: 37. doi: 10.3389/fbioe.2014.00037
[4]	E. R. Andreeva, E. G. Rudimov, A. N. Gornostaeva, V. I. Beklemyshev, Makhonin, II, U. O. Maugeri and L. B. Buravkova (2013): In vitro study of interactions between silicon-containing nanoparticles and human peripheral blood leukocytes. Bull Exp Biol Med, 155: 396-8. doi: https://www.ncbi.nlm.nih.gov/pubmed/24137611
[5]	M. Aoyama, K. Hata, K. Higashisaka, K. Nagano, Y. Yoshioka and Y. Tsutsumi (2016): Clusterin in the protein corona plays a key role in the stealth effect of nanoparticles against phagocytes. Biochem Biophys Res Commun, 480: 690-695. doi: 10.1016/j.bbrc.2016.10.121
[6]	M. Aoyama, Y. Yoshioka, Y. Arai, H. Hirai, R. Ishimoto, K. Nagano, K. Higashisaka, T. Nagai and Y. Tsutsumi (2017): Intracellular trafficking of particles inside endosomal vesicles is regulated by particle size. J Control Release, 260: 183-193. doi: 10.1016/j.jconrel.2017.06.007
[7]	A. Aranda, L. Sequedo, L. Tolosa, G. Quintas, E. Burello, J. V. Castell and L. Gombau (2013): Dichloro-dihydro-fluorescein diacetate (DCFH-DA) assay: a quantitative method for oxidative stress assessment of nanoparticle-treated cells. Toxicol In Vitro, 27: 954-63. doi: 10.1016/j.tiv.2013.01.016
[8]	C. O. Asweto, J. Wu, H. Hu, L. Feng, X. Yang, J. Duan and Z. Sun (2017): Combined Effect of Silica Nanoparticles and Benzo[a]pyrene on Cell Cycle Arrest Induction and Apoptosis in Human Umbilical Vein Endothelial Cells. Int J Environ Res Public Health, 14: doi: 10.3390/ijerph14030289
[9]	J. Athinarayanan, V. S. Periasamy, M. A. Alsaif, A. A. Al-Warthan and A. A. Alshatwi (2014): Presence of nanosilica (E551) in commercial food products: TNF-mediated oxidative stress and altered cell cycle progression in human lung fibroblast cells. Cell Biol Toxicol, 30: 89-100. doi: 10.1007/s10565-014-9271-8
[10]	X. Bao, X. Wei, Y. Wang, H. Jiang, D. Yu and M. Hu (2014): Effect of silica-based nanomaterials and their derivate with PEGylation on cementoblasts. Ann Biomed Eng, 42: 1781-9. doi: 10.1007/s10439-014-1012-x
[11]	D. Battal, A. Celik, G. Guler, A. Aktas, S. Yildirimcan, K. Ocakoglu and U. Comelekoglu (2015): SiO2 Nanoparticule-induced size-dependent genotoxicity - an in vitro study using sister chromatid exchange, micronucleus and comet assay. Drug Chem Toxicol, 38: 196-204. doi: 10.3109/01480545.2014.928721
[12]	J. M. Berg, A. A. Romoser, D. E. Figueroa, C. Spencer West and C. M. Sayes (2013): Comparative cytological responses of lung epithelial and pleural mesothelial cells following in vitro exposure to nanoscale SiO2. Toxicol In Vitro, 27: 24-33. doi: 10.1016/j.tiv.2012.09.002
[13]	A. Bermejo-Nogales, M. L. Fernandez-Cruz and J. M. Navas (2017): Fish cell lines as a tool for the ecotoxicity assessment and ranking of engineered nanomaterials. Regul Toxicol Pharmacol, 90: 297-307. doi: 10.1016/j.yrtph.2017.09.029
[14]	M. Best, G. Phillips, C. Fowler, J. Rowland and J. Elsom (2015): Characterisation and cytotoxic screening of metal oxide nanoparticles putative of interest to oral healthcare formulations in non-keratinised human oral mucosa cells in vitro. Toxicol In Vitro, 30: 402-11. doi: 10.1016/j.tiv.2015.09.022
[15]	K. Bhattacharya, G. Kilic, P. M. Costa and B. Fadeel (2017): Cytotoxicity screening and cytokine profiling of nineteen nanomaterials enables hazard ranking and grouping based on

	inflammogenic potential. Nanotoxicology, 11: 809-826. doi: 10.1080/17435390.2017.1363309
[16]	J. Blechinger, A. T. Bauer, A. A. Torrano, C. Gorzelanny, C. Brauchle and S. W. Schneider (2013): Uptake kinetics and nanotoxicity of silica nanoparticles are cell type dependent. Small, 9: 3970-80, 3906. doi: 10.1002/smll.201301004
[17]	C. Brandenberger, N. L. Rowley, D. N. Jackson-Humbles, Q. Zhang, L. A. Bramble, R. P. Lewandowski, J. G. Wagner, W. Chen, B. L. Kaplan, N. E. Kaminski, G. L. Baker, R. M. Worden and J. R. Harkema (2013): Engineered silica nanoparticles act as adjuvants to enhance allergic airway disease in mice. Part Fibre Toxicol, 10: 26. doi: 10.1186/1743-8977-10-26
[18]	D. Breznan, D. Das, C. MacKinnon-Roy, B. Simard, P. Kumarathasan and R. Vincent (2015): Non-specific interaction of carbon nanotubes with the resazurin assay reagent: impact on in vitro assessment of nanoparticle cytotoxicity. Toxicol In Vitro, 29: 142-7. doi: 10.1016/j.tiv.2014.09.009
[19]	D. Breznan, D. D. Das, J. S. O'Brien, C. MacKinnon-Roy, S. Nimesh, N. Q. Vuong, S. Bernatchez, N. DeSilva, M. Hill, P. Kumarathasan and R. Vincent (2017): Differential cytotoxic and inflammatory potency of amorphous silicon dioxide nanoparticles of similar size in multiple cell lines. Nanotoxicology, 11: 223-235. doi: 10.1080/17435390.2017.1287313
[20]	D. M. Brown, H. Johnston, E. Gubbins and V. Stone (2014): Serum enhanced cytokine responses of macrophages to silica and iron oxide particles and nanomaterials: a comparison of serum to lung lining fluid and albumin dispersions. J Appl Toxicol, 34: 1177-87. doi: 10.1002/jat.2998
[21]	D. M. Brown, N. Kanase, B. Gaiser, H. Johnston and V. Stone (2014): Inflammation and gene expression in the rat lung after instillation of silica nanoparticles: effect of size, dispersion medium and particle surface charge. Toxicol Lett, 224: 147-56. doi: http://dx.doi.org/10.1016/j.toxlet.2013.10.019
[22]	W. Cao, Y. Zhou, Y. Niu, X. Zhu, Y. Song and R. Guo (2017): Quantitative Analysis of Hepatic Toxicity in Rats Induced by Inhalable Silica Nanoparticles Using Acoustic Radiation Force Imaging. J Ultrasound Med, 36: 1829-1839. doi: 10.1002/jum.14219
[23]	W. T. Chan, C. C. Liu, J. S. Chiang Chiau, S. T. Tsai, C. K. Liang, M. L. Cheng, H. C. Lee, C. Y. Yeung and S. Y. Hou (2017): In vivo toxicologic study of larger silica nanoparticles in mice. Int J Nanomedicine, 12: 3421-3432. doi: 10.2147/IJN.S126823
[24]	Q. Chen, Y. Xue and J. Sun (2013): Kupffer cell-mediated hepatic injury induced by silica nanoparticles in vitro and in vivo. Int J Nanomedicine, 8: 1129-40. doi: 10.2147/IJN.S42242
[25]	S. L. Chia and D. T. Leong (2016): Reducing ZnO nanoparticles toxicity through silica coating. Heliyon, 2: e00177. doi: 10.1016/j.heliyon.2016.e00177
[26]	W. S. Cho, R. Duffin, M. Bradley, I. L. Megson, W. MacNee, J. K. Lee, J. Jeong and K. Donaldson (2013): Predictive value of in vitro assays depends on the mechanism of toxicity of metal oxide nanoparticles. Part Fibre Toxicol, 10: 55. doi: 10.1186/1743-8977-10-55
[27]	S. Y. Choi, N. Yang, S. K. Jeon and T. H. Yoon (2014): Semi-quantitative estimation of cellular SiO2 nanoparticles using flow cytometry combined with X-ray fluorescence measurements. Cytometry A, 85: 771-80. doi: 10.1002/cyto.a.22481
[28]	V. Christen, M. Camenzind and K. Fent (2014): Silica nanoparticles induce endoplasmic reticulum stress response, oxidative stress and activate the mitogen-activated protein kinase (MAPK) signaling pathway. Toxicol Rep, 1: 1143-1151. doi: 10.1016/j.toxrep.2014.10.023

[29]	V. Christen and K. Fent (2016): Silica nanoparticles induce endoplasmic reticulum stress response and activate mitogen activated kinase (MAPK) signalling. Toxicol Rep, 3: 832-840. doi: 10.1016/j.toxrep.2016.10.009
[30]	C. Contado, J. Mejia, O. Lozano Garcia, J. P. Piret, E. Dumortier, O. Toussaint and S. Lucas (2016): Physicochemical and toxicological evaluation of silica nanoparticles suitable for food and consumer products collected by following the EC recommendation. Anal Bioanal Chem, 408: 271-86. doi: 10.1007/s00216-015-9101-8
[31]	S. Corradi, E. Dakou, A. Yadav, L. C. Thomassen, M. Kirsch-Volders and L. Leyns (2015): Morphological observation of embryoid bodies completes the in vitro evaluation of nanomaterial embryotoxicity in the embryonic stem cell test (EST). Toxicol In Vitro, 29: 1587-96. doi: 10.1016/j.tiv.2015.06.015
[32]	S. Correia Carreira, L. Walker, K. Paul and M. Saunders (2015): The toxicity, transport and uptake of nanoparticles in the in vitro BeWo b30 placental cell barrier model used within NanoTEST. Nanotoxicology, 9 Suppl 1: 66-78. doi: 10.3109/17435390.2013.833317
[33]	B. Dalzon, C. Aude-Garcia, V. Collin-Faure, H. Diemer, D. Beal, F. Dussert, D. Fenel, G. Schoehn, S. Cianferani, M. Carriere and T. Rabilloud (2017): Differential proteomics highlights macrophage-specific responses to amorphous silica nanoparticles. Nanoscale, 9: 9641-9658. doi: 10.1039/c7nr02140b
[34]	M. Das, D. K. Yi and S. S. An (2015): Analyses of protein corona on bare and silica-coated gold nanorods against four mammalian cells. Int J Nanomedicine, 10: 1521-45. doi: 10.2147/IJN.S76187
[35]	D. C. Davidson, R. Derk, X. He, T. A. Stueckle, J. Cohen, S. V. Pirela, P. Demokritou, Y. Rojanasakul and L. Wang (2016): Direct stimulation of human fibroblasts by nCeO2 in vitro is attenuated with an amorphous silica coating. Part Fibre Toxicol, 13: 23. doi: 10.1186/s12989-016-0134-8
[36]	N. Decan, D. Wu, A. Williams, S. Bernatchez, M. Johnston, M. Hill and S. Halappanavar (2016): Characterization of in vitro genotoxic, cytotoxic and transcriptomic responses following exposures to amorphous silica of different sizes. Mutat Res Genet Toxicol Environ Mutagen, 796: 8-22. doi: 10.1016/j.mrgentox.2015.11.011
[37]	M. Delaval, S. Boland, B. Solhonne, M. A. Nicola, S. Mornet, A. Baeza-Squiban, J. M. Sallenave and I. Garcia-Verdugo (2015): Acute exposure to silica nanoparticles enhances mortality and increases lung permeability in a mouse model of Pseudomonas aeruginosa pneumonia. Part Fibre Toxicol, 12: 1. doi: 10.1186/s12989-014-0078-9
[38]	G. DeLoid, B. Casella, S. Pirela, R. Filoramo, G. Pyrgiotakis, P. Demokritou and L. Kobzik (2016): Effects of engineered nanomaterial exposure on macrophage innate immune function. NanoImpact, 2: 70-81. doi: 10.1016/j.impact.2016.07.001
[39]	E. Demir, S. Aksakal, F. Turna, B. Kaya and R. Marcos (2015): In vivo genotoxic effects of four different nano-sizes forms of silica nanoparticles in Drosophila melanogaster. J Hazard Mater, 283: 260-6. doi: 10.1016/j.jhazmat.2014.09.029
[40]	E. Demir and V. Castranova (2016): Genotoxic effects of synthetic amorphous silica nanoparticles in the mouse lymphoma assay. Toxicol Rep, 3: 807-815. doi: 10.1016/j.toxrep.2016.10.006
[41]	L. Di Cristo, D. Movia, M. G. Bianchi, M. Allegri, B. M. Mohamed, A. P. Bell, C. Moore, S. Pinelli, K. Rasmussen, J. Riego-Sintes, A. Prina-Mello, O. Bussolati and E. Bergamaschi (2016): Proinflammatory Effects of Pyrogenic and Precipitated Amorphous Silica Nanoparticles in Innate Immunity Cells. Toxicol Sci, 150: 40-53. doi: 10.1093/toxsci/kfv258

[42]	D. Docter, C. Bantz, D. Westmeier, H. J. Galla, Q. Wang, J. C. Kirkpatrick, P. Nielsen, M. Maskos and R. H. Stauber (2014): The protein corona protects against size- and dose-dependent toxicity of amorphous silica nanoparticles. Beilstein J Nanotechnol, 5: 1380-92. doi: 10.3762/bjnano.5.151
[43]	M. D. Driessen, S. Mues, A. Vennemann, B. Hellack, A. Bannuscher, V. Vimalakanthan, C. Riebeling, R. Ossig, M. Wiemann, J. Schnekenburger, T. A. Kuhlbusch, B. Renard, A. Luch and A. Haase (2015): Proteomic analysis of protein carbonylation: a useful tool to unravel nanoparticle toxicity mechanisms. Part Fibre Toxicol, 12: 36. doi: 10.1186/s12989-015-0108-2
[44]	Z. Du, D. Zhao, L. Jing, G. Cui, M. Jin, Y. Li, X. Liu, Y. Liu, H. Du, C. Guo, X. Zhou and Z. Sun (2013): Cardiovascular toxicity of different sizes amorphous silica nanoparticles in rats after intratracheal instillation. Cardiovasc Toxicol, 13: 194-207. doi: 10.1007/s12012-013-9198-y
[45]	Z. J. Du, G. Q. Cui, J. Zhang, X. M. Liu, Z. H. Zhang, Q. Jia, J. C. Ng, C. Peng, C. X. Bo and H. Shao (2017): Inhibition of gap junction intercellular communication is involved in silica nanoparticles-induced H9c2 cardiomyocytes apoptosis via the mitochondrial pathway. Int J Nanomedicine, 12: 2179-2188. doi: 10.2147/IJN.S127904
[46]	J. Duan, H. Hu, L. Feng, X. Yang and Z. Sun (2017): Silica nanoparticles inhibit macrophage activity and angiogenesis via VEGFR2-mediated MAPK signaling pathway in zebrafish embryos. Chemosphere, 183: 483-490. doi: 10.1016/j.chemosphere.2017.05.138
[47]	J. Duan, H. Hu, Q. Li, L. Jiang, Y. Zou, Y. Wang and Z. Sun (2016): Combined toxicity of silica nanoparticles and methylmercury on cardiovascular system in zebrafish (Danio rerio) embryos. Environ Toxicol Pharmacol, 44: 120-7. doi: 10.1016/j.etap.2016.05.004
[48]	J. Duan, V. K. Kodali, M. J. Gaffrey, J. Guo, R. K. Chu, D. G. Camp, R. D. Smith, B. D. Thrall and W. J. Qian (2016): Quantitative Profiling of Protein S-Glutathionylation Reveals Redox-Dependent Regulation of Macrophage Function during Nanoparticle-Induced Oxidative Stress. ACS Nano, 10: 524-38. doi: 10.1021/acsnano.5b05524
[49]	J. Duan, S. Liang, Y. Yu, Y. Li, L. Wang, Z. Wu, Y. Chen, M. R. Miller and Z. Sun (2018): Inflammation-coagulation response and thrombotic effects induced by silica nanoparticles in zebrafish embryos. Nanotoxicology, 1-15. doi: 10.1080/17435390.2018.1461267
[50]	J. Duan, Y. Yu, Y. Li, Y. Li, H. Liu, L. Jing, M. Yang, J. Wang, C. Li and Z. Sun (2016): Low-dose exposure of silica nanoparticles induces cardiac dysfunction via neutrophil-mediated inflammation and cardiac contraction in zebrafish embryos. Nanotoxicology, 10: 575-85. doi: 10.3109/17435390.2015.1102981
[51]	J. Duan, Y. Yu, Y. Li, Y. Wang and Z. Sun (2016): Inflammatory response and blood hypercoagulable state induced by low level co-exposure with silica nanoparticles and benzo[a]pyrene in zebrafish (Danio rerio) embryos. Chemosphere, 151: 152-62. doi: 10.1016/j.chemosphere.2016.02.079
[52]	J. Duan, Y. Yu, Y. Li, Y. Yu, Y. Li, X. Zhou, P. Huang and Z. Sun (2013): Toxic effect of silica nanoparticles on endothelial cells through DNA damage response via Chk1-dependent G2/M checkpoint. PLoS One, 8: e62087. doi: 10.1371/journal.pone.0062087
[53]	J. Duan, Y. Yu, Y. Li, Y. Yu and Z. Sun (2013): Cardiovascular toxicity evaluation of silica nanoparticles in endothelial cells and zebrafish model. Biomaterials, 34: 5853-62. doi: 10.1016/j.biomaterials.2013.04.032
[54]	J. Duan, Y. Yu, H. Shi, L. Tian, C. Guo, P. Huang, X. Zhou, S. Peng and Z. Sun (2013): Toxic effects of silica nanoparticles on zebrafish embryos and larvae. PLoS One, 8: e74606. doi: 10.1371/journal.pone.0074606

[55]	J. Duan, Y. Yu, Y. Yu, Y. Li, P. Huang, X. Zhou, S. Peng and Z. Sun (2014): Silica nanoparticles enhance autophagic activity, disturb endothelial cell homeostasis and impair angiogenesis. Part Fibre Toxicol, 11: 50. doi: 10.1186/s12989-014-0050-8
[56]	J. Duan, Y. Yu, Y. Yu, Y. Li, J. Wang, W. Geng, L. Jiang, Q. Li, X. Zhou and Z. Sun (2014): Silica nanoparticles induce autophagy and endothelial dysfunction via the PI3K/Akt/mTOR signaling pathway. Int J Nanomedicine, 9: 5131-41. doi: 10.2147/IJN.S71074
[57]	V. Dubes, T. Parpaite, T. Ducret, J. F. Quignard, S. Mornet, N. Reinhardt, I. Baudrimont, M. Dubois, V. Freund-Michel, R. Marthan, B. Muller, J. P. Savineau and A. Courtois (2017): Calcium signalling induced by in vitro exposure to silicium dioxide nanoparticles in rat pulmonary artery smooth muscle cells. Toxicology, 375: 37-47. doi: 10.1016/j.tox.2016.12.002
[58]	A. D. Ducray, A. Felser, J. Zielinski, A. Bittner, J. V. Burgi, J. M. Nuoffer, M. Frenz and M. Mevissen (2017): Effects of silica nanoparticle exposure on mitochondrial function during neuronal differentiation. J Nanobiotechnology, 15: 49. doi: 10.1186/s12951-017-0284-3
[59]	A. D. Ducray, A. Stojiljkovic, A. Moller, M. H. Stoffel, H. R. Widmer, M. Frenz and M. Mevissen (2017): Uptake of silica nanoparticles in the brain and effects on neuronal differentiation using different in vitro models. Nanomedicine, 13: 1195-1204. doi: 10.1016/j.nano.2016.11.001
[60]	E. Dumitrescu, D. P. Karunaratne, M. K. Prochaska, X. Liu, K. N. Wallace and S. Andreescu (2017): Developmental toxicity of glycine-coated silica nanoparticles in embryonic zebrafish. Environ Pollut, 229: 439-447. doi: 10.1016/j.envpol.2017.06.016
[61]	L. Farcal, F. Torres Andon, L. Di Cristo, B. M. Rotoli, O. Bussolati, E. Bergamaschi, A. Mech, N. B. Hartmann, K. Rasmussen, J. Riego-Sintes, J. Ponti, A. Kinsner-Ovaskainen, F. Rossi, A. Oomen, P. Bos, R. Chen, R. Bai, C. Chen, L. Rocks, N. Fulton, B. Ross, G. Hutchison, L. Tran, S. Mues, R. Ossig, J. Schnekenburger, L. Campagnolo, L. Vecchione, A. Pietroiusti and B. Fadeel (2015): Comprehensive In Vitro Toxicity Testing of a Panel of Representative Oxide Nanomaterials: First Steps towards an Intelligent Testing Strategy. PLoS One, 10: e0127174. doi: 10.1371/journal.pone.0127174
[62]	L. R. Farcal, C. Uboldi, D. Mehn, G. Giudetti, P. Nativo, J. Ponti, D. Gilliland, F. Rossi and A. Bal-Price (2013): Mechanisms of toxicity induced by SiO2 nanoparticles of in vitro human alveolar barrier: effects on cytokine production, oxidative stress induction, surfactant proteins A mRNA expression and nanoparticles uptake. Nanotoxicology, 7: 1095-110. doi: 10.3109/17435390.2012.710658
[63]	C. Fede, C. Millino, B. Pacchioni, B. Celegato, C. Compagnin, P. Martini, F. Selvestrel, F. Mancin, L. Celotti, G. Lanfranchi, M. Mognato and S. Cagnin (2014): Altered gene transcription in human cells treated with Ludox(R) silica nanoparticles. Int J Environ Res Public Health, 11: 8867-90. doi: 10.3390/ijerph110908867
[64]	C. Fedeli, F. Selvestrel, R. Tavano, D. Segat, F. Mancin and E. Papini (2013): Catastrophic inflammatory death of monocytes and macrophages by overtaking of a critical dose of endocytosed synthetic amorphous silica nanoparticles/serum protein complexes. Nanomedicine (Lond), 8: 1101-26. doi: 10.2217/nnm.12.136
[65]	W. Feng, J. Guo, H. Huang, B. Xia, H. Liu, J. Li, S. Lin, T. Li, J. Liu and H. Li (2015): Human normal bronchial epithelial cells: a novel in vitro cell model for toxicity evaluation. PLoS One, 10: e0123520. doi: 10.1371/journal.pone.0123520
[66]	S. Ferchichi, H. Trabelsi, I. Azzouz, A. Hanini, A. Rejeb, O. Tebourbi, M. Sakly and H. Abdelmelek (2016): Evaluation of oxidative response and tissular damage in rat lungs

	exposed to silica-coated gold nanoparticles under static magnetic fields. Int J Nanomedicine, 11: 2711-9. doi: 10.2147/IJN.S103140
[67]	N. Fernandez-Bertolez, C. Costa, F. Brandao, G. Kilic, J. A. Duarte, J. P. Teixeira, E. Pasaro, V. Valdiglesias and B. Laffon (2018): Toxicological assessment of silica-coated iron oxide nanoparticles in human astrocytes. Food Chem Toxicol, 118: 13-23. doi: 10.1016/j.fct.2018.04.058
[68]	D. Ferraro, U. Anselmi-Tamburini, I. G. Tredici, V. Ricci and P. Sommi (2016): Overestimation of nanoparticles-induced DNA damage determined by the comet assay. Nanotoxicology, 10: 861-70. doi: 10.3109/17435390.2015.1130274
[69]	N. L. Flaherty, A. Chandrasekaran, M. del Pilar Sosa Pena, G. A. Roth, S. A. Brenner, T. J. Begley and J. A. Melendez (2015): Comparative analysis of redox and inflammatory properties of pristine nanomaterials and commonly used semiconductor manufacturing nano-abrasives. Toxicol Lett, 239: 205-15. doi: 10.1016/j.toxlet.2015.09.025
[70]	R. Foldbjerg, J. Wang, C. Beer, K. Thorsen, D. S. Sutherland and H. Autrup (2013): Biological effects induced by BSA-stabilized silica nanoparticles in mammalian cell lines. Chem Biol Interact, 204: 28-38. doi: 10.1016/j.cbi.2013.04.007
[71]	C. Fontana, A. Kirsch, C. Seidel, L. Marpeaux, C. Darne, L. Gate, A. Remy and Y. Guichard (2017): In vitro cell transformation induced by synthetic amorphous silica nanoparticles. Mutat Res, 823: 22-27. doi: 10.1016/j.mrgentox.2017.08.002
[72]	C. Freese, D. Schreiner, L. Anspach, C. Bantz, M. Maskos, R. E. Unger and C. J. Kirkpatrick (2014): In vitro investigation of silica nanoparticle uptake into human endothelial cells under physiological cyclic stretch. Part Fibre Toxicol, 11: 68. doi: 10.1186/s12989-014-0068-y
[73]	K. Fujioka, S. Hanada, Y. Inoue, K. Sato, K. Hirakuri, K. Shiraishi, F. Kanaya, K. Ikeda, R. Usui, K. Yamamoto, S. U. Kim and Y. Manome (2014): Effects of silica and titanium oxide particles on a human neural stem cell line: morphology, mitochondrial activity, and gene expression of differentiation markers. Int J Mol Sci, 15: 11742-59. doi: 10.3390/ijms150711742
[74]	C. Gambardella, S. Morgana, G. D. Bari, P. Ramoino, M. Bramini, A. Diaspro, C. Falugi and M. Faimali (2015): Multidisciplinary screening of toxicity induced by silica nanoparticles during sea urchin development. Chemosphere, 139: 486-95. doi: 10.1016/j.chemosphere.2015.07.072
[75]	A. Garcia-Rodriguez, L. Vila, C. Cortes, A. Hernandez and R. Marcos (2018): Exploring the usefulness of the complex in vitro intestinal epithelial model Caco-2/HT29/Raji-B in nanotoxicology. Food Chem Toxicol, 113: 162-170. doi: 10.1016/j.fct.2018.01.042
[76]	H. Gehrke, A. Fruhmesser, J. Pelka, M. Esselen, L. L. Hecht, H. Blank, H. P. Schuchmann, D. Gerthsen, C. Marquardt, S. Diabate, C. Weiss and D. Marko (2013): In vitro toxicity of amorphous silica nanoparticles in human colon carcinoma cells. Nanotoxicology, 7: 274-93. doi: 10.3109/17435390.2011.652207
[77]	I. George, S. Vranic, S. Boland, A. Courtois and A. Baeza-Squiban (2015): Development of an in vitro model of human bronchial epithelial barrier to study nanoparticle translocation. Toxicol In Vitro, 29: 51-8. doi: 10.1016/j.tiv.2014.08.003
[78]	A. Gilardino, F. Catalano, F. A. Ruffinatti, G. Alberto, B. Nilius, S. Antoniotti, G. Martra and D. Lovisolo (2015): Interaction of SiO2 nanoparticles with neuronal cells: Ionic mechanisms involved in the perturbation of calcium homeostasis. Int J Biochem Cell Biol, 66: 101-11. doi: 10.1016/j.biocel.2015.07.012

[79]	G. Giovannini, P. Warncke, D. Fischer, O. Stranik, A. J. Hall and V. Gubala (2018): Improving colloidal stability of silica nanoparticles when stored in responsive gel: application and toxicity study. Nanotoxicology, 1-16. doi: 10.1080/17435390.2018.1457729
[80]	C. Gong, L. Yang, J. Zhou, X. Guo and Z. Zhuang (2017): Possible role of PAPR-1 in protecting human HaCaT cells against cytotoxicity of SiO2 nanoparticles. Toxicol Lett, 280: 213-221. doi: 10.1016/j.toxlet.2017.07.213
[81]	L. Gonzalez, M. De Santis Puzzonia, R. Ricci, F. Aureli, G. Guarguaglini, F. Cubadda, L. Leyns, E. Cundari and M. Kirsch-Volders (2015): Amorphous silica nanoparticles alter microtubule dynamics and cell migration. Nanotoxicology, 9: 729-36. doi: 10.3109/17435390.2014.969791
[82]	L. Gonzalez, M. Lukamowicz-Rajska, L. C. Thomassen, C. E. Kirschhock, L. Leyns, D. Lison, J. A. Martens, A. Elhajouji and M. Kirsch-Volders (2014): Co-assessment of cell cycle and micronucleus frequencies demonstrates the influence of serum on the in vitro genotoxic response to amorphous monodisperse silica nanoparticles of varying sizes. Nanotoxicology, 8: 876-84. doi: 10.3109/17435390.2013.842266
[83]	R. Guadagnini, K. Moreau, S. Hussain, F. Marano and S. Boland (2015): Toxicity evaluation of engineered nanoparticles for medical applications using pulmonary epithelial cells. Nanotoxicology, 9 Suppl 1: 25-32. doi: 10.3109/17435390.2013.855830
[84]	C. E. Guerrero-Beltran, J. Bernal-Ramirez, O. Lozano, Y. Oropeza-Almazan, E. C. Castillo, J. R. Garza, N. Garcia, J. Vela, A. Garcia-Garcia, E. Ortega, G. Torre-Amione, N. Ornelas-Soto and G. Garcia-Rivas (2017): Silica nanoparticles induce cardiotoxicity interfering with energetic status and Ca(2+) handling in adult rat cardiomyocytes. Am J Physiol Heart Circ Physiol, 312: H645-H661. doi: 10.1152/ajpheart.00564.2016
[85]	Y. Guichard, C. Fontana, E. Chavinier, F. Terzetti, L. Gate, S. Binet and C. Darne (2016): Cytotoxic and genotoxic evaluation of different synthetic amorphous silica nanomaterials in the V79 cell line. Toxicol Ind Health, 32: 1639-50. doi: 10.1177/0748233715572562
[86]	Y. Guichard, M. A. Maire, S. Sebillaud, C. Fontana, C. Langlais, J. C. Micillino, C. Darne, J. Roszak, M. Stepnik, V. Fessard, S. Binet and L. Gate (2015): Genotoxicity of synthetic amorphous silica nanoparticles in rats following short-term exposure. Part 2: intratracheal instillation and intravenous injection. Environ Mol Mutagen, 56: 228-44. doi: 10.1002/em.21928
[87]	C. Guo, R. Ma, X. Liu, T. Chen, Y. Li, Y. Yu, J. Duan, X. Zhou, Y. Li and Z. Sun (2018): Silica nanoparticles promote oxLDL-induced macrophage lipid accumulation and apoptosis via endoplasmic reticulum stress signaling. Sci Total Environ, 631-632: 570-579. doi: 10.1016/j.scitotenv.2018.02.312
[88]	C. Guo, J. Wang, L. Jing, R. Ma, X. Liu, L. Gao, L. Cao, J. Duan, X. Zhou, Y. Li and Z. Sun (2018): Mitochondrial dysfunction, perturbations of mitochondrial dynamics and biogenesis involved in endothelial injury induced by silica nanoparticles. Environ Pollut, 236: 926-936. doi: 10.1016/j.envpol.2017.10.060
[89]	C. Guo, J. Wang, M. Yang, Y. Li, S. Cui, X. Zhou, Y. Li and Z. Sun (2017): Amorphous silica nanoparticles induce malignant transformation and tumorigenesis of human lung epithelial cells via P53 signaling. Nanotoxicology, 11: 1176-1194. doi: 10.1080/17435390.2017.1403658
[90]	C. Guo, Y. Xia, P. Niu, L. Jiang, J. Duan, Y. Yu, X. Zhou, Y. Li and Z. Sun (2015): Silica nanoparticles induce oxidative stress, inflammation, and endothelial dysfunction in vitro via activation of the MAPK/Nrf2 pathway and nuclear factor-kappaB signaling. Int J Nanomedicine, 10: 1463-77. doi: 10.2147/IJN.S76114

[91]	C. Guo, M. Yang, L. Jing, J. Wang, Y. Yu, Y. Li, J. Duan, X. Zhou, Y. Li and Z. Sun (2016): Amorphous silica nanoparticles trigger vascular endothelial cell injury through apoptosis and autophagy via reactive oxygen species-mediated MAPK/Bcl-2 and PI3K/Akt/mTOR signaling. Int J Nanomedicine, 11: 5257-5276. doi: 10.2147/IJN.S112030
[92]	Z. Guo, N. J. Martucci, Y. Liu, E. Yoo, E. Tako and G. J. Mahler (2018): Silicon dioxide nanoparticle exposure affects small intestine function in an in vitro model. Nanotoxicology, 1-24. doi: 10.1080/17435390.2018.1463407
[93]	M. Guttenberg, L. Bezerra, N. M. Neu-Baker, M. Del Pilar Sosa Idelchik, A. Elder, G. Oberdorster and S. A. Brenner (2016): Biodistribution of inhaled metal oxide nanoparticles mimicking occupational exposure: a preliminary investigation using enhanced darkfield microscopy. J Biophotonics, 9: 987-993. doi: 10.1002/jbio.201600125
[94]	S. W. Ha, C. E. Camalier, M. N. Weitzmann, G. R. Beck, Jr. and J. K. Lee (2013): Long-Term Monitoring of the Physicochemical Properties of Silica-Based Nanoparticles on the Rate of Endocytosis and Exocytosis and Consequences of Cell Division. Soft Mater, 11: 195-203. doi: 10.1080/1539445X.2012.617641
[95]	A. Haase, N. Dommershausen, M. Schulz, R. Landsiedel, P. Reichardt, B. C. Krause, J. Tentschert and A. Luch (2017): Genotoxicity testing of different surface-functionalized SiO2, ZrO2 and silver nanomaterials in 3D human bronchial models. Arch Toxicol, 91: 3991-4007. doi: 10.1007/s00204-017-2015-9
[96]	N. Haberl, S. Hirn, M. Holzer, G. Zuchtriegel, M. Rehberg and F. Krombach (2015): Effects of acute systemic administration of TiO2, ZnO, SiO2, and Ag nanoparticles on hemodynamics, hemostasis and leukocyte recruitment. Nanotoxicology, 9: 963-71. doi: 10.3109/17435390.2014.992815
[97]	B. Halamoda Kenzaoui, C. Chapuis Bernasconi and L. Juillerat-Jeanneret (2013): Stress reaction of kidney epithelial cells to inorganic solid-core nanoparticles. Cell Biol Toxicol, 29: 39-58. doi: 10.1007/s10565-012-9236-8
[98]	H. Han, Y. H. Park, H. J. Park, K. Lee, K. Um, J. W. Park and J. H. Lee (2016): Toxic and adjuvant effects of silica nanoparticles on ovalbumin-induced allergic airway inflammation in mice. Respir Res, 17: 60. doi: 10.1186/s12931-016-0376-x
[99]	T. Handa, T. Hirai, N. Izumi, S. I. Eto, S. I. Tsunoda, K. Nagano, K. Higashisaka, Y. Yoshioka and Y. Tsutsumi (2017): Identifying a size-specific hazard of silica nanoparticles after intravenous administration and its relationship to the other hazards that have negative correlations with the particle size in mice. Nanotechnology, 28: 135101. doi: 10.1088/1361-6528/aa5d7c
[100]	I. Hansjosten, J. Rapp, L. Reiner, R. Vatter, S. Fritsch-Decker, R. Peravali, T. Palosaari, E. Joossens, K. Gerloff, P. Macko, M. Whelan, D. Gilliland, I. Ojea-Jimenez, M. P. Monopoli, L. Rocks, D. Garry, K. Dawson, P. J. F. Rottgermann, A. Murschhauser, J. O. Radler, S. V. Y. Tang, P. Gooden, M. A. Belinga-Desaunay, A. O. Khan, S. Briffa, E. Guggenheim, A. Papadiamantis, I. Lynch, E. Valsami-Jones, S. Diabate and C. Weiss (2018): Microscopy-based high-throughput assays enable multi-parametric analysis to assess adverse effects of nanomaterials in various cell lines. Arch Toxicol, 92: 633-649. doi: 10.1007/s00204-017-2106-7
[101]	M. Hashimoto and S. Imazato (2015): Cytotoxic and genotoxic characterization of aluminum and silicon oxide nanoparticles in macrophages. Dent Mater, 31: 556-64. doi: 10.1016/j.dental.2015.02.009
[102]	R. Hassankhani, M. Esmaeillou, A. A. Tehrani, K. Nasirzadeh, F. Khadir and H. Maadi (2015): In vivo toxicity of orally administrated silicon dioxide nanoparticles in healthy adult mice. Environ Sci Pollut Res Int, 22: 1127-32. doi: 10.1007/s11356-014-3413-7

[103]	H. L. Herd, K. T. Bartlett, J. A. Gustafson, L. D. McGill and H. Ghandehari (2015): Macrophage silica nanoparticle response is phenotypically dependent. Biomaterials, 53: 574-82. doi: 10.1016/j.biomaterials.2015.02.070
[104]	T. Hirai, Y. Yoshioka, N. Izumi, K. Ichihashi, T. Handa, N. Nishijima, E. Uemura, K. Sagami, H. Takahashi, M. Yamaguchi, K. Nagano, Y. Mukai, H. Kamada, S. Tsunoda, K. J. Ishii, K. Higashisaka and Y. Tsutsumi (2016): Metal nanoparticles in the presence of lipopolysaccharides trigger the onset of metal allergy in mice. Nat Nanotechnol, 11: 808-16. doi: 10.1038/nnano.2016.88
[105]	T. Hirai, Y. Yoshioka, H. Takahashi, K. Ichihashi, A. Udaka, T. Mori, N. Nishijima, T. Yoshida, K. Nagano, H. Kamada, S. Tsunoda, T. Takagi, K. J. Ishii, H. Nabeshi, T. Yoshikawa, K. Higashisaka and Y. Tsutsumi (2015): Cutaneous exposure to agglomerates of silica nanoparticles and allergen results in IgE-biased immune response and increased sensitivity to anaphylaxis in mice. Part Fibre Toxicol, 12: 16. doi: 10.1186/s12989-015-0095-3
[106]	F. Hofmann, R. Blasche, M. Kasper and K. Barth (2015): A co-culture system with an organotypic lung slice and an immortal alveolar macrophage cell line to quantify silica-induced inflammation. PLoS One, 10: e0117056. doi: 10.1371/journal.pone.0117056
[107]	T. Hofmann, S. Schneider, A. Wolterbeek, H. van de Sandt, R. Landsiedel and B. van Ravenzwaay (2015): Prenatal toxicity of synthetic amorphous silica nanomaterial in rats. Reprod Toxicol, 56: 141-6. doi: 10.1016/j.reprotox.2015.04.006
[108]	J. Hoppstadter, M. Seif, A. Dembek, C. Cavelius, H. Huwer, A. Kraegeloh and A. K. Kiemer (2015): M2 polarization enhances silica nanoparticle uptake by macrophages. Front Pharmacol, 6: 55. doi: 10.3389/fphar.2015.00055
[109]	M. Horie, K. Nishio, H. Kato, S. Endoh, K. Fujita, A. Nakamura, Y. Hagihara, Y. Yoshida and H. Iwahashi (2014): Evaluation of cellular effects of silicon dioxide nanoparticles. Toxicol Mech Methods, 24: 196-203. doi: 10.3109/15376516.2013.879505
[110]	M. Horie, M. Stowe, M. Tabei and E. Kuroda (2015): Pharyngeal aspiration of metal oxide nanoparticles showed potential of allergy aggravation effect to inhaled ovalbumin. Inhal Toxicol, 27: 181-90. doi: 10.3109/08958378.2015.1026618
[111]	C. S. Hughes, L. M. Colhoun, B. K. Bains, J. D. Kilgour, R. E. Burden, J. F. Burrows, E. C. Lavelle, B. F. Gilmore and C. J. Scott (2016): Extracellular cathepsin S and intracellular caspase 1 activation are surrogate biomarkers of particulate-induced lysosomal disruption in macrophages. Part Fibre Toxicol, 13: 19. doi: 10.1186/s12989-016-0129-5
[112]	S. Imai, Y. Yoshioka, Y. Morishita, T. Yoshida, M. Uji, K. Nagano, Y. Mukai, H. Kamada, S. Tsunoda, K. Higashisaka and Y. Tsutsumi (2014): Size and surface modification of amorphous silica particles determine their effects on the activity of human CYP3A4 in vitro. Nanoscale Res Lett, 9: 651. doi: 10.1186/1556-276X-9-651
[113]	A. Irfan, M. Cauchi, W. Edmands, N. J. Gooderham, J. Njuguna and H. Zhu (2014): Assessment of temporal dose-toxicity relationship of fumed silica nanoparticle in human lung A549 cells by conventional cytotoxicity and (1)H-NMR-based extracellular metabonomic assays. Toxicol Sci, 138: 354-64. doi: 10.1093/toxsci/kfu009
[114]	K. Isoda, E. Tetsuka, Y. Shimizu, K. Saitoh, I. Ishida and M. Tezuka (2013): Liver injury induced by thirty- and fifty-nanometer-diameter silica nanoparticles. Biol Pharm Bull, 36: 370-5. doi: https://doi.org/10.1248/bpb.b12-00738
[115]	A. Ivask, T. Titma, M. Visnapuu, H. Vija, A. Kakinen, M. Sihtmae, S. Pokhrel, L. Madler, M. Heinlaan, V. Kisand, R. Shimmo and A. Kahru (2015): Toxicity of 11 Metal Oxide

	Nanoparticles to Three Mammalian Cell Types In Vitro. Curr Top Med Chem, 15: 1914-29. doi, https://www.ncbi.nlm.nih.gov/pubmed/25961521
[116]	E. Izak-Nau, K. Kenesei, K. Murali, M. Voetz, S. Eiden, V. F. Puntes, A. Duschl and E. Madarasz (2014): Interaction of differently functionalized fluorescent silica nanoparticles with neural stem- and tissue-type cells. Nanotoxicology, 8 Suppl 1: 138-48. doi: 10.3109/17435390.2013.864427
[117]	S. Jatana, B. C. Palmer, S. J. Phelan and L. A. DeLouise (2017): Immunomodulatory Effects of Nanoparticles on Skin Allergy. Sci Rep, 7: 3979. doi: 10.1038/s41598-017-03729-2
[118]	D. Jeon, H. Kim, K. Nam, S. Oh, S. H. Son and I. Shin (2017): Cytotoxic Effect of Nano-SiO2 in Human Breast Cancer Cells via Modulation of EGFR Signaling Cascades. Anticancer Res, 37: 6189-6197. doi: 10.21873/anticanres.12068
[119]	L. Jiang, Y. Yu, Y. Li, Y. Yu, J. Duan, Y. Zou, Q. Li and Z. Sun (2016): Oxidative Damage and Energy Metabolism Disorder Contribute to the Hemolytic Effect of Amorphous Silica Nanoparticles. Nanoscale Res Lett, 11: 57. doi: 10.1186/s11671-016-1280-5
[120]	J. Jimenez-Villarreal, D. I. Rivas-Armendariz, R. D. Arellano Perez-Vertti, E. Olivas Calderon, R. Garcia-Garza, N. D. Betancourt-Martinez, L. B. Serrano-Gallardo and J. Moran-Martinez (2017): Relationship between lymphocyte DNA fragmentation and dose of iron oxide (Fe2O3) and silicon oxide (SiO2) nanoparticles. Genet Mol Res, 16: doi: 10.4238/gmr16019206
[121]	G. Karunakaran, R. Suriyaprabha, V. Rajendran and N. Kannan (2015): Effect of contact angle, zeta potential and particles size on the in vitro studies of Al2O3 and SiO2 nanoparticles. IET Nanobiotechnol, 9: 27-34. doi: 10.1049/iet-nbt.2013.0067
[122]	J. Kasper, M. I. Hermanns, C. Bantz, O. Koshkina, T. Lang, M. Maskos, C. Pohl, R. E. Unger and C. J. Kirkpatrick (2013): Interactions of silica nanoparticles with lung epithelial cells and the association to flotillins. Arch Toxicol, 87: 1053-65. doi: 10.1007/s00204-012-0876-5
[123]	J. Y. Kasper, L. Feiden, M. I. Hermanns, C. Bantz, M. Maskos, R. E. Unger and C. J. Kirkpatrick (2015): Pulmonary surfactant augments cytotoxicity of silica nanoparticles: Studies on an in vitro air-blood barrier model. Beilstein J Nanotechnol, 6: 517-28. doi: 10.3762/bjnano.6.54
[124]	A. Katsumiti, I. Arostegui, M. Oron, D. Gilliland, E. Valsami-Jones and M. P. Cajaraville (2016): Cytotoxicity of Au, ZnO and SiO(2) NPs using in vitro assays with mussel hemocytes and gill cells: Relevance of size, shape and additives. Nanotoxicology, 10: 185-93. doi: 10.3109/17435390.2015.1039092
[125]	A. Kermanizadeh, K. Jantzen, D. M. Brown, P. Moller and S. Loft (2018): A Flow Cytometry-based Method for the Screening of Nanomaterial-induced Reactive Oxygen Species Production in Leukocytes Subpopulations in Whole Blood. Basic Clin Pharmacol Toxicol, 122: 149-156. doi: 10.1111/bcpt.12845
[126]	H. Kettiger, D. Sen Karaman, L. Schiesser, J. M. Rosenholm and J. Huwyler (2015): Comparative safety evaluation of silica-based particles. Toxicol In Vitro, 30: 355-63. doi: 10.1016/j.tiv.2015.09.030
[127]	I. Y. Kim, E. Joachim, H. Choi and K. Kim (2015): Toxicity of silica nanoparticles depends on size, dose, and cell type. Nanomedicine, 11: 1407-16. doi: 10.1016/j.nano.2015.03.004
[128]	J. Kim, Y. J. Heo and S. Shin (2016): Haemocompatibility evaluation of silica nanomaterials using hemorheological measurements. Clin Hemorheol Microcirc, 62: 99-107. doi: 10.3233/CH-151953

[129]	J. E. Kim, H. Kim, S. S. An, E. H. Maeng, M. K. Kim and Y. J. Song (2014): In vitro cytotoxicity of SiO2 or ZnO nanoparticles with different sizes and surface charges on U373MG human glioblastoma cells. Int J Nanomedicine, 9 Suppl 2: 235-41. doi: 10.2147/IJN.S57936
[130]	J. H. Kim, C. S. Kim, R. M. Ignacio, D. H. Kim, M. E. Sajo, E. H. Maeng, X. F. Qi, S. E. Park, Y. R. Kim, M. K. Kim, K. J. Lee and S. K. Kim (2014): Immunotoxicity of silicon dioxide nanoparticles with different sizes and electrostatic charge. Int J Nanomedicine, 9 Suppl 2: 183-93. doi: 10.2147/IJN.S57934
[131]	J. Y. Kim, J. H. Park, M. Kim, H. Jeong, J. Hong, R. S. Chuck and C. Y. Park (2017): Safety of Nonporous Silica Nanoparticles in Human Corneal Endothelial Cells. Sci Rep, 7: 14566. doi: 10.1038/s41598-017-15247-2
[132]	M. Kim, J. H. Park, H. Jeong, J. Hong, W. S. Choi, B. H. Lee and C. Y. Park (2017): An Evaluation of the in vivo Safety of Nonporous Silica Nanoparticles: Ocular Topical Administration versus Oral Administration. Sci Rep, 7: 8238. doi: 10.1038/s41598-017-08843-9
[133]	M. K. Kim, J. A. Lee, M. R. Jo and S. J. Choi (2016): Bioavailability of Silica, Titanium Dioxide, and Zinc Oxide Nanoparticles in Rats. J Nanosci Nanotechnol, 16: 6580-6. doi: 10.1166/jnn.2016.12350
[134]	Y. H. Kim, E. Boykin, T. Stevens, K. Lavrich and M. I. Gilmour (2014): Comparative lung toxicity of engineered nanomaterials utilizing in vitro, ex vivo and in vivo approaches. J Nanobiotechnology, 12: 47. doi: 10.1186/s12951-014-0047-3
[135]	Y. R. Kim, S. Y. Lee, E. J. Lee, S. H. Park, N. W. Seong, H. S. Seo, S. S. Shin, S. J. Kim, E. H. Meang, M. K. Park, M. S. Kim, C. S. Kim, S. K. Kim, S. W. Son, Y. R. Seo, B. H. Kang, B. S. Han, S. S. An, B. J. Lee and M. K. Kim (2014): Toxicity of colloidal silica nanoparticles administered orally for 90 days in rats. Int J Nanomedicine, 9 Suppl 2: 67-78. doi: 10.2147/IJN.S57925
[136]	K. T. Kitchin, S. Stirdivant, B. L. Robinette, B. T. Castellon and X. Liang (2017): Metabolomic effects of CeO2, SiO2 and CuO metal oxide nanomaterials on HepG2 cells. Part Fibre Toxicol, 14: 50. doi: 10.1186/s12989-017-0230-4
[137]	F. Koch, A. M. Moller, M. Frenz, U. Pieles, K. Kuehni-Boghenbor and M. Mevissen (2014): An in vitro toxicity evaluation of gold-, PLLA- and PCL-coated silica nanoparticles in neuronal cells for nanoparticle-assisted laser-tissue soldering. Toxicol In Vitro, 28: 990-8. doi: 10.1016/j.tiv.2014.04.010
[138]	V. Kodali, M. H. Littke, S. C. Tilton, J. G. Teeguarden, L. Shi, C. W. Frevert, W. Wang, J. G. Pounds and B. D. Thrall (2013): Dysregulation of macrophage activation profiles by engineered nanoparticles. ACS Nano, 7: 6997-7010. doi: 10.1021/nn402145t
[139]	S. Kojima, Y. Negishi, M. Tsukimoto, T. Takenouchi, H. Kitani and K. Takeda (2014): Purinergic signaling via P2X7 receptor mediates IL-1beta production in Kupffer cells exposed to silica nanoparticle. Toxicology, 321: 13-20. doi: 10.1016/j.tox.2014.03.008
[140]	S. N. Kolle, U. G. Sauer, M. C. Moreno, W. Teubner, W. Wohlleben and R. Landsiedel (2016): Eye irritation testing of nanomaterials using the EpiOcular eye irritation test and the bovine corneal opacity and permeability assay. Part Fibre Toxicol, 13: 18. doi: 10.1186/s12989-016-0128-6
[141]	N. V. Konduru, R. J. Jimenez, A. Swami, S. Friend, V. Castranova, P. Demokritou, J. D. Brain and R. M. Molina (2015): Silica coating influences the corona and biokinetics of cerium oxide nanoparticles. Part Fibre Toxicol, 12: 31. doi: 10.1186/s12989-015-0106-4

[142]	N. V. Konduru, K. M. Murdaugh, G. A. Sotiriou, T. C. Donaghey, P. Demokritou, J. D. Brain and R. M. Molina (2014): Bioavailability, distribution and clearance of tracheally-instilled and gavaged uncoated or silica-coated zinc oxide nanoparticles. Part Fibre Toxicol, 11: 44. doi: 10.1186/s12989-014-0044-6
[143]	V. Kononenko, A. Erman, T. Petan, I. Krizaj, S. Kralj, D. Makovec and D. Drobne (2017): Harmful at non-cytotoxic concentrations: SiO2-SPIONs affect surfactant metabolism and lamellar body biogenesis in A549 human alveolar epithelial cells. Nanotoxicology, 11: 419-429. doi: 10.1080/17435390.2017.1309704
[144]	A. I. Kozelskaya, A. V. Panin, I. A. Khlusov, P. V. Mokrushnikov, B. N. Zaitsev, D. I. Kuzmenko and G. Y. Vasyukov (2016): Morphological changes of the red blood cells treated with metal oxide nanoparticles. Toxicol In Vitro, 37: 34-40. doi: 10.1016/j.tiv.2016.08.012
[145]	R. Kretowski, M. Kusaczuk, M. Naumowicz, J. Kotynska, B. Szynaka and M. Cechowska-Pasko (2017): The Effects of Silica Nanoparticles on Apoptosis and Autophagy of Glioblastoma Cell Lines. Nanomaterials (Basel), 7: doi: 10.3390/nano7080230
[146]	K. Krishna Priya, M. Ramesh, M. Saravanan and N. Ponpandian (2015): Ecological risk assessment of silicon dioxide nanoparticles in a freshwater fish Labeo rohita: Hematology, ionoregulation and gill Na(+)/K(+) ATPase activity. Ecotoxicol Environ Saf, 120: 295-302. doi: 10.1016/j.ecoenv.2015.05.032
[147]	D. Kumar, I. Mutreja, P. C. Keshvan, M. Bhat, A. K. Dinda and S. Mitra (2015): Organically Modified Silica Nanoparticles Interaction with Macrophage Cells: Assessment of Cell Viability on the Basis of Physicochemical Properties. J Pharm Sci, 104: 3943-51. doi: 10.1002/jps.24614
[148]	A. Kurtz-Chalot, C. Villiers, J. Pourchez, D. Boudard, M. Martini, P. N. Marche, M. Cottier and V. Forest (2017): Impact of silica nanoparticle surface chemistry on protein corona formation and consequential interactions with biological cells. Mater Sci Eng C Mater Biol Appl, 75: 16-24. doi: 10.1016/j.msec.2017.02.028
[149]	M. Kusaczuk, R. Kretowski, M. Naumowicz, A. Stypulkowska and M. Cechowska-Pasko (2018): Silica nanoparticle-induced oxidative stress and mitochondrial damage is followed by activation of intrinsic apoptosis pathway in glioblastoma cells. Int J Nanomedicine, 13: 2279-2294. doi: 10.2147/IJN.S158393
[150]	J. Y. Kwon, H. L. Kim, J. Y. Lee, Y. H. Ju, J. S. Kim, S. H. Kang, Y. R. Kim, J. K. Lee, J. Jeong, M. K. Kim, E. H. Maeng and Y. R. Seo (2014): Undetactable levels of genotoxicity of SiO2 nanoparticles in in vitro and in vivo tests. Int J Nanomedicine, 9 Suppl 2: 173-81. doi: 10.2147/IJN.S57933
[151]	J. M. Lacave, A. Retuerto, U. Vicario-Pares, D. Gilliland, M. Oron, M. P. Cajaraville and A. Orbea (2016): Effects of metal-bearing nanoparticles (Ag, Au, CdS, ZnO, SiO2) on developing zebrafish embryos. Nanotechnology, 27: 325102. doi: 10.1088/0957-4484/27/32/325102
[152]	M. Lag, T. Skuland, A. Godymchuk, T. H. T. Nguyen, H. L. T. Pham and M. Refsnes (2018): Silica Nanoparticle-induced Cytokine Responses in BEAS-2B and HBEC3-KT Cells: Significance of Particle Size and Signalling Pathways in Different Lung Cell Cultures. Basic Clin Pharmacol Toxicol, doi: 10.1111/bcpt.12963
[153]	L. Landgraf, D. Nordmeyer, P. Schmiel, Q. Gao, S. Ritz, S. G. J, S. Grass, S. Diabate, L. Treuel, C. Graf, E. Ruhl, K. Landfester, V. Mailander, C. Weiss, R. Zellner and I. Hilger (2017): Validation of weak biological effects by round robin experiments: cytotoxicity/biocompatibility of SiO2 and polymer nanoparticles in HepG2 cells. Sci Rep, 7: 4341. doi: 10.1038/s41598-017-02958-9

[154]	R. Landsiedel, L. Ma-Hock, T. Hofmann, M. Wiemann, V. Strauss, S. Treumann, W. Wohlleben, S. Groters, K. Wiench and B. van Ravenzwaay (2014): Application of short-term inhalation studies to assess the inhalation toxicity of nanomaterials. Part Fibre Toxicol, 11: 16. doi: 10.1186/1743-8977-11-16
[155]	A. Lankoff, M. Arabski, A. Wegierek-Ciuk, M. Kruszewski, H. Lisowska, A. Banasik-Nowak, K. Rozga-Wijas, M. Wojewodzka and S. Slomkowski (2013): Effect of surface modification of silica nanoparticles on toxicity and cellular uptake by human peripheral blood lymphocytes in vitro. Nanotoxicology, 7: 235-50. doi: 10.3109/17435390.2011.649796
[156]	S. F. Larner, J. Wang, J. Goodman, M. B. O. Altman, M. Xin and K. K. W. Wang (2017): In Vitro Neurotoxicity Resulting from Exposure of Cultured Neural Cells to Several Types of Nanoparticles. J Cell Death, 10: 1179670717694523. doi: 10.1177/1179670717694523
[157]	L. Leclerc, J. P. Klein, V. Forest, D. Boudard, M. Martini, J. Pourchez, M. G. Blanchin and M. Cottier (2015): Testicular biodistribution of silica-gold nanoparticles after intramuscular injection in mice. Biomed Microdevices, 17: 66. doi: 10.1007/s10544-015-9968-3
[158]	S. Lee, M. S. Kim, D. Lee, T. K. Kwon, D. Khang, H. S. Yun and S. H. Kim (2013): The comparative immunotoxicity of mesoporous silica nanoparticles and colloidal silica nanoparticles in mice. Int J Nanomedicine, 8: 147-58. doi: 10.2147/IJN.S39534
[159]	S. E. Lehman, A. S. Morris, P. S. Mueller, A. K. Salem, V. H. Grassian and S. C. Larsen (2016): Silica Nanoparticle-Generated ROS as a Predictor of Cellular Toxicity: Mechanistic Insights and Safety by Design. Environ Sci Nano, 3: 56-66. doi: 10.1039/C5EN00179J
[160]	M. Leppanen, A. Korpi, S. Mikkonen, P. Yli-Pirila, M. Lehto, L. Pylkkanen, H. Wolff, V. M. Kosma, H. Alenius, J. Joutsensaari and P. Pasanen (2015): Inhaled silica-coated TiO2 nanoparticles induced airway irritation, airflow limitation and inflammation in mice. Nanotoxicology, 9: 210-8. doi: 10.3109/17435390.2014.914260
[161]	X. Li, B. Liu, X. L. Li, Y. X. Li, M. Z. Sun, D. Y. Chen, X. Zhao and X. Z. Feng (2014): SiO2 nanoparticles change colour preference and cause Parkinson's-like behaviour in zebrafish. Sci Rep, 4: 3810. doi: 10.1038/srep03810
[162]	C. L. Liang, Q. Xiang, W. M. Cui, J. Fang, N. N. Sun, X. P. Zhang, Y. N. Li, H. Yang, Z. Yu and X. D. Jia (2018): Subchronic Oral Toxicity of Silica Nanoparticles and Silica Microparticles in Rats. Biomed Environ Sci, 31: 197-207. doi: 10.3967/bes2018.025
[163]	H. Liang, C. Jin, Y. Tang, F. Wang, C. Ma and Y. Yang (2014): Cytotoxicity of silica nanoparticles on HaCaT cells. J Appl Toxicol, 34: 367-72. doi: 10.1002/jat.2953
[164]	C. Lin, X. Zhao, D. Sun, L. Zhang, W. Fang, T. Zhu, Q. Wang, B. Liu, S. Wei, G. Chen, Z. Xu and X. Gao (2016): Transcriptional activation of follistatin by Nrf2 protects pulmonary epithelial cells against silica nanoparticle-induced oxidative stress. Sci Rep, 6: 21133. doi: 10.1038/srep21133
[165]	Z. Lin, L. Ma, Z. G. X, H. Zhang and B. Lin (2013): A comparative study of lung toxicity in rats induced by three types of nanomaterials. Nanoscale Res Lett, 8: 521. doi: 10.1186/1556-276X-8-521
[166]	J. Liu, M. Yang, L. Jing, L. Ren, J. Wei, J. Zhang, F. Zhang, J. Duan, X. Zhou and Z. Sun (2018): Silica nanoparticle exposure inducing granulosa cell apoptosis and follicular atresia in female Balb/c mice. Environ Sci Pollut Res Int, 25: 3423-3434. doi: 10.1007/s11356-017-0724-5
[167]	T. Liu, H. Liu, C. Fu, L. Li, D. Chen, Y. Zhang and F. Tang (2013): Smaller silica nanorattles reabsorbed by intestinal aggravate multiple organs damage. J Nanosci Nanotechnol, 13: 6506-16. doi: 10.1166/jnn.2013.7545

[168]	W. Liu, T. Hu, L. Zhou, D. Wu, X. Huang, X. Ren, Y. Lv, W. Hong, G. Huang, Z. Lin and J. Liu (2017): Nrf2 protects against oxidative stress induced by SiO2 nanoparticles. Nanomedicine (Lond), 12: 2303-2318. doi: 10.2217/nnm-2017-0046
[169]	J. Lojk, S. Prpar Mihevc, V. B. Bregar, M. Pavlin and B. Rogelj (2017): The Effect of Different Types of Nanoparticles on FUS and TDP-43 Solubility and Subcellular Localization. Neurotox Res, 32: 325-339. doi: 10.1007/s12640-017-9734-9
[170]	C. F. Lu, L. Z. Li, W. Zhou, J. Zhao, Y. M. Wang and S. Q. Peng (2017): Silica nanoparticles and lead acetate co-exposure triggered synergistic cytotoxicity in A549 cells through potentiation of mitochondria-dependent apoptosis induction. Environ Toxicol Pharmacol, 52: 114-120. doi: 10.1016/j.etap.2017.04.001
[171]	C. F. Lu, X. Y. Yuan, L. Z. Li, W. Zhou, J. Zhao, Y. M. Wang and S. Q. Peng (2015): Combined exposure to nano-silica and lead induced potentiation of oxidative stress and DNA damage in human lung epithelial cells. Ecotoxicol Environ Saf, 122: 537-44. doi: 10.1016/j.ecoenv.2015.09.030
[172]	X. Lu, C. Ji, T. Jin and X. Fan (2015): The effects of size and surface modification of amorphous silica particles on biodistribution and liver metabolism in mice. Nanotechnology, 26: 175101. doi: 10.1088/0957-4484/26/17/175101
[173]	X. Lu, T. Jin, Y. Jin, L. Wu, B. Hu, Y. Tian and X. Fan (2013): Toxicogenomic analysis of the particle dose- and size-response relationship of silica particles-induced toxicity in mice. Nanotechnology, 24: 015106. doi: 10.1088/0957-4484/24/1/015106
[174]	J. Ma, R. R. Mercer, M. Barger, D. Schwegler-Berry, J. M. Cohen, P. Demokritou and V. Castranova (2015): Effects of amorphous silica coating on cerium oxide nanoparticles induced pulmonary responses. Toxicol Appl Pharmacol, 288: 63-73. doi: 10.1016/j.taap.2015.07.012
[175]	M. A. Malvindi, V. De Matteis, A. Galeone, V. Brunetti, G. C. Anyfantis, A. Athanassiou, R. Cingolani and P. P. Pompa (2014): Toxicity assessment of silica coated iron oxide nanoparticles and biocompatibility improvement by surface engineering. PLoS One, 9: e85835. doi: 10.1371/journal.pone.0085835
[176]	C. Marquardt, S. Fritsch-Decker, M. Al-Rawi, S. Diabate and C. Weiss (2017): Autophagy induced by silica nanoparticles protects RAW264.7 macrophages from cell death. Toxicology, 379: 40-47. doi: 10.1016/j.tox.2017.01.019
[177]	A. Marucco, E. Gazzano, D. Ghigo, E. Enrico and I. Fenoglio (2016): Fibrinogen enhances the inflammatory response of alveolar macrophages to TiO2, SiO2 and carbon nanomaterials. Nanotoxicology, 10: 1-9. doi: 10.3109/17435390.2014.978405
[178]	V. Marzaioli, C. J. Gross, I. Weichenmeier, C. B. Schmidt-Weber, J. Gutermuth, O. Gross and F. Alessandrini (2017): Specific Surface Modifications of Silica Nanoparticles Diminish Inflammasome Activation and In Vivo Expression of Selected Inflammatory Genes. Nanomaterials (Basel), 7: doi: 10.3390/nano7110355
[179]	E. Maser, M. Schulz, U. G. Sauer, M. Wiemann, L. Ma-Hock, W. Wohlleben, A. Hartwig and R. Landsiedel (2015): In vitro and in vivo genotoxicity investigations of differently sized amorphous SiO2 nanomaterials. Mutat Res Genet Toxicol Environ Mutagen, 794: 57-74. doi: 10.1016/j.mrgentox.2015.10.005
[180]	G. H. Mathisen, V. Ansteinsson, J. T. Samuelsen, R. Becher, J. E. Dahl and A. K. Bolling (2015): TEGDMA and filler particles from dental composites additively attenuate LPS-induced cytokine release from the macrophage cell line RAW 264.7. Clin Oral Investig, 19: 61-9. doi: 10.1007/s00784-014-1212-7

[181]	K. Matsuo, S. Hirobe, N. Okada and S. Nakagawa (2016): Analysis of Skin Permeability and Toxicological Properties of Amorphous Silica Particles. Biol Pharm Bull, 39: 1201-5. doi: 10.1248/bpb.b16-00258
[182]	C. McCracken, A. Zane, D. A. Knight, P. K. Dutta and W. J. Waldman (2013): Minimal intestinal epithelial cell toxicity in response to short- and long-term food-relevant inorganic nanoparticle exposure. Chem Res Toxicol, 26: 1514-25. doi: 10.1021/tx400231u
[183]	C. McCracken, A. Zane, D. A. Knight, E. Hommel, P. K. Dutta and W. J. Waldman (2015): Oxidative stress-mediated inhibition of intestinal epithelial cell proliferation by silver nanoparticles. Toxicol In Vitro, 29: 1793-808. doi: 10.1016/j.tiv.2015.07.017
[184]	C. Meindl, T. Kueznik, M. Bosch, E. Roblegg and E. Frohlich (2015): Intracellular calcium levels as screening tool for nanoparticle toxicity. J Appl Toxicol, 35: 1150-9. doi: 10.1002/jat.3160
[185]	A. Mendoza, J. A. Torres-Hernandez, J. G. Ault, J. H. Pedersen-Lane, D. Gao and D. A. Lawrence (2014): Silica nanoparticles induce oxidative stress and inflammation of human peripheral blood mononuclear cells. Cell Stress Chaperones, 19: 777-90. doi: 10.1007/s12192-014-0502-y
[186]	C. Messerschmidt, D. Hofmann, A. Kroeger, K. Landfester, V. Mailander and I. Lieberwirth (2016): On the pathway of cellular uptake: new insight into the interaction between the cell membrane and very small nanoparticles. Beilstein J Nanotechnol, 7: 1296-1311. doi: 10.3762/bjnano.7.121
[187]	A. S. Morris, A. Adamcakova-Dodd, S. E. Lehman, A. Wongrakpanich, P. S. Thorne, S. C. Larsen and A. K. Salem (2016): Amine modification of nonporous silica nanoparticles reduces inflammatory response following intratracheal instillation in murine lungs. Toxicol Lett, 241: 207-15. doi: 10.1016/j.toxlet.2015.11.006
[188]	N. P. Mortensen, G. B. Hurst, W. Wang, C. M. Foster, P. D. Nallathamby and S. T. Retterer (2013): Dynamic development of the protein corona on silica nanoparticles: composition and role in toxicity. Nanoscale, 5: 6372-80. doi: 10.1039/c3nr33280b
[189]	M. Mrakovcic, C. Meindl, E. Roblegg and E. Frohlich (2014): Reaction of monocytes to polystyrene and silica nanoparticles in short-term and long-term exposures. Toxicol Res (Camb), 3: 86-97. doi: 10.1039/C3TX50112D
[190]	J. Mytych, M. Wnuk and S. I. Rattan (2016): Low doses of nanodiamonds and silica nanoparticles have beneficial hormetic effects in normal human skin fibroblasts in culture. Chemosphere, 148: 307-15. doi: 10.1016/j.chemosphere.2016.01.045
[191]	C. Nagakura, Y. Negishi, M. Tsukimoto, S. Itou, T. Kondo, K. Takeda and S. Kojima (2014): Involvement of P2Y11 receptor in silica nanoparticles 30-induced IL-6 production by human keratinocytes. Toxicology, 322: 61-8. doi: 10.1016/j.tox.2014.03.010
[192]	T. Nagano, K. Higashisaka, A. Kunieda, Y. Iwahara, K. Tanaka, K. Nagano, Y. Abe, H. Kamada, S. Tsunoda, H. Nabeshi, T. Yoshikawa, Y. Yoshioka and Y. Tsutsumi (2013): Liver-specific microRNAs as biomarkers of nanomaterial-induced liver damage. Nanotechnology, 24: 405102. doi: 10.1088/0957-4484/24/40/405102
[193]	T. Nagano, K. Nagano, H. Nabeshi, T. Yoshida, H. Kamada, S. I. Tsunoda, J. Q. Gao, K. Higashisaka, Y. Yoshioka and Y. Tsutsumi (2017): Modifying the Surface of Silica Nanoparticles with Amino or Carboxyl Groups Decreases Their Cytotoxicity to Parenchymal Hepatocytes. Biol Pharm Bull, 40: 726-728. doi: 10.1248/bpb.b16-00917
[194]	K. Nakanishi, M. Tsukimoto, S. Tanuma, K. Takeda and S. Kojima (2016): Silica nanoparticles activate purinergic signaling via P2X7 receptor in dendritic cells, leading to

	production of pro-inflammatory cytokines. Toxicol In Vitro, 35: 202-11. doi: 10.1016/j.tiv.2016.06.003
[195]	A. Nemmar, S. Albarwani, S. Beegam, P. Yuvaraju, J. Yasin, S. Attoub and B. H. Ali (2014): Amorphous silica nanoparticles impair vascular homeostasis and induce systemic inflammation. Int J Nanomedicine, 9: 2779-89. doi: 10.2147/IJN.S52818
[196]	A. Nemmar, P. Yuvaraju, S. Beegam, J. Yasin, R. A. Dhaheri, M. A. Fahim and B. H. Ali (2015): In vitro platelet aggregation and oxidative stress caused by amorphous silica nanoparticles. Int J Physiol Pathophysiol Pharmacol, 7: 27-33. doi, https://www.ncbi.nlm.nih.gov/pubmed/26069526
[197]	A. Nemmar, P. Yuvaraju, S. Beegam, J. Yasin, E. E. Kazzam and B. H. Ali (2016): Oxidative stress, inflammation, and DNA damage in multiple organs of mice acutely exposed to amorphous silica nanoparticles. Int J Nanomedicine, 11: 919-28. doi: 10.2147/IJN.S92278
[198]	N. Nishijima, T. Hirai, K. Misato, M. Aoyama, E. Kuroda, K. J. Ishii, K. Higashisaka, Y. Yoshioka and Y. Tsutsumi (2017): Human Scavenger Receptor A1-Mediated Inflammatory Response to Silica Particle Exposure Is Size Specific. Front Immunol, 8: 379. doi: 10.3389/fimmu.2017.00379
[199]	Y. Niu, W. I. Chan, N. Yu, J. Gan, L. Dong and C. Wang (2015): APTES-modified nanosilica--but neither APTES nor nanosilica--inhibits endothelial cell growth via arrest of cell cycle at G1 phase. J Biomater Appl, 30: 608-17. doi: 10.1177/0885328215598497
[200]	Y. M. Niu, X. L. Zhu, B. Chang, Z. H. Tong, W. Cao, P. H. Qiao, L. Y. Zhang, J. Zhao and Y. G. Song (2016): Nanosilica and Polyacrylate/Nanosilica: A Comparative Study of Acute Toxicity. Biomed Res Int, 2016: 9353275. doi: 10.1155/2016/9353275
[201]	J. S. Nowak, D. Mehn, P. Nativo, C. P. Garcia, S. Gioria, I. Ojea-Jimenez, D. Gilliland and F. Rossi (2014): Silica nanoparticle uptake induces survival mechanism in A549 cells by the activation of autophagy but not apoptosis. Toxicol Lett, 224: 84-92. doi: 10.1016/j.toxlet.2013.10.003
[202]	I. Ojea-Jimenez, P. Urban, F. Barahona, M. Pedroni, R. Capomaccio, G. Ceccone, A. Kinsner-Ovaskainen, F. Rossi and D. Gilliland (2016): Highly Flexible Platform for Tuning Surface Properties of Silica Nanoparticles and Monitoring Their Biological Interaction. ACS Appl Mater Interfaces, 8: 4838-50. doi: 10.1021/acsami.5b11216
[203]	O. Okoturo-Evans, A. Dybowska, E. Valsami-Jones, J. Cupitt, M. Gierula, A. R. Boobis and R. J. Edwards (2013): Elucidation of toxicity pathways in lung epithelial cells induced by silicon dioxide nanoparticles. PLoS One, 8: e72363. doi: 10.1371/journal.pone.0072363
[204]	A. Onodera, K. Yayama, H. Morosawa, Y. Ishii, Y. Tsutsumi and Y. Kawai (2017): Reduction of calcium flux from the extracellular region and endoplasmic reticulum by amorphous nano-silica particles owing to carboxy group addition on their surface. Biochem Biophys Rep, 9: 330-334. doi: 10.1016/j.bbrep.2017.01.014
[205]	A. Onodera, K. Yayama, A. Tanaka, H. Morosawa, T. Furuta, N. Takeda, K. Kakiguchi, S. Yonemura, I. Yanagihara, Y. Tsutsumi and Y. Kawai (2016): Amorphous nanosilica particles evoke vascular relaxation through PI3K/Akt/eNOS signaling. Fundam Clin Pharmacol, 30: 419-28. doi: 10.1111/fcp.12206
[206]	A. Panas, A. Comouth, H. Saathoff, T. Leisner, M. Al-Rawi, M. Simon, G. Seemann, O. Dossel, S. Mulhopt, H. R. Paur, S. Fritsch-Decker, C. Weiss and S. Diabate (2014): Silica nanoparticles are less toxic to human lung cells when deposited at the air-liquid interface compared to conventional submerged exposure. Beilstein J Nanotechnol, 5: 1590-1602. doi: 10.3762/bjnano.5.171

[207]	A. Panas, C. Marquardt, O. Nalcaci, H. Bockhorn, W. Baumann, H. R. Paur, S. Mulhopt, S. Diabate and C. Weiss (2013): Screening of different metal oxide nanoparticles reveals selective toxicity and inflammatory potential of silica nanoparticles in lung epithelial cells and macrophages. Nanotoxicology, 7: 259-73. doi: 10.3109/17435390.2011.652206
[208]	A. Pandey, S. Chandra, L. K. Chauhan, G. Narayan and D. K. Chowdhuri (2013): Cellular internalization and stress response of ingested amorphous silica nanoparticles in the midgut of Drosophila melanogaster. Biochim Biophys Acta, 1830: 2256-66. doi: 10.1016/j.bbagen.2012.10.001
[209]	M. Pardo, T. Shuster-Meiseles, S. Levin-Zaidman, A. Rudich and Y. Rudich (2014): Low cytotoxicity of inorganic nanotubes and fullerene-like nanostructures in human bronchial epithelial cells: relation to inflammatory gene induction and antioxidant response. Environ Sci Technol, 48: 3457-66. doi: 10.1021/es500065z
[210]	H. J. Park, J. H. Sohn, Y. J. Kim, Y. H. Park, H. Han, K. H. Park, K. Lee, H. Choi, K. Um, I. H. Choi, J. W. Park and J. H. Lee (2015): Acute exposure to silica nanoparticles aggravate airway inflammation: different effects according to surface characteristics. Exp Mol Med, 47: e173. doi: 10.1038/emm.2015.50
[211]	J. H. Park, H. Jeong, J. Hong, M. Chang, M. Kim, R. S. Chuck, J. K. Lee and C. Y. Park (2016): The Effect of Silica Nanoparticles on Human Corneal Epithelial Cells. Sci Rep, 6: 37762. doi: 10.1038/srep37762
[212]	A. Parveen, S. H. Rizvi, Sushma, F. Mahdi, I. Ahmad, P. P. Singh and A. A. Mahdi (2017): Intranasal exposure to silica nanoparticles induces alterations in pro-inflammatory environment of rat brain. Toxicol Ind Health, 33: 119-132. doi: 10.1177/0748233715602985
[213]	P. Pellen-Mussi, S. Tricot-Doleux, C. Neaime, N. Nerambourg, F. Cabello-Hurtado, S. Cordier, F. Grasset and S. Jeanne (2018): Evaluation of Functional SiO(2) Nanoparticles Toxicity by a 3D Culture Model. J Nanosci Nanotechnol, 18: 3148-3157. doi: 10.1166/jnn.2018.14619
[214]	V. S. Periasamy, J. Athinarayanan, M. A. Akbarsha and A. A. Alshatwi (2015): Silica nanoparticles induced metabolic stress through EGR1, CCND, and E2F1 genes in human mesenchymal stem cells. Appl Biochem Biotechnol, 175: 1181-92. doi: 10.1007/s12010-014-1342-z
[215]	S. N. Petrache Voicu, D. Dinu, C. Sima, A. Hermenean, A. Ardelean, E. Codrici, M. S. Stan, O. Zarnescu and A. Dinischiotu (2015): Silica Nanoparticles Induce Oxidative Stress and Autophagy but Not Apoptosis in the MRC-5 Cell Line. Int J Mol Sci, 16: 29398-416. doi: 10.3390/ijms161226171
[216]	L. Petrick, M. Rosenblat, N. Paland and M. Aviram (2016): Silicon dioxide nanoparticles increase macrophage atherogenicity: Stimulation of cellular cytotoxicity, oxidative stress, and triglycerides accumulation. Environ Toxicol, 31: 713-23. doi: 10.1002/tox.22084
[217]	S. Pfuhler, T. R. Downs, A. J. Allemang, Y. Shan and M. E. Crosby (2017): Weak silica nanomaterial-induced genotoxicity can be explained by indirect DNA damage as shown by the OGG1-modified comet assay and genomic analysis. Mutagenesis, 32: 5-12. doi: 10.1093/mutage/gew064
[218]	D. H. Pham, B. De Roo, X. B. Nguyen, M. Vervaele, A. Kecskes, A. Ny, D. Copmans, H. Vriens, J. P. Locquet, P. Hoet and P. A. de Witte (2016): Use of Zebrafish Larvae as a Multi-Endpoint Platform to Characterize the Toxicity Profile of Silica Nanoparticles. Sci Rep, 6: 37145. doi: 10.1038/srep37145
[219]	G. Phukan, T. H. Shin, J. S. Shim, M. J. Paik, J. K. Lee, S. Choi, Y. M. Kim, S. H. Kang, H. S. Kim, Y. Kang, S. H. Lee, M. M. Mouradian and G. Lee (2016): Silica-coated magnetic

	nanoparticles impair proteasome activity and increase the formation of cytoplasmic inclusion bodies in vitro. Sci Rep, 6: 29095. doi: 10.1038/srep29095
[220]	A. Pietroiusti, L. Vecchione, M. A. Malvindi, C. Aru, M. Massimiani, A. Camaioni, A. Magrini, R. Bernardini, S. Sabella, P. P. Pompa and L. Campagnolo (2018): Relevance to investigate different stages of pregnancy to highlight toxic effects of nanoparticles: The example of silica. Toxicol Appl Pharmacol, 342: 60-68. doi: 10.1016/j.taap.2018.01.026
[221]	C. Pisani, J. C. Gaillard, V. Nouvel, M. Odorico, J. Armengaud and O. Prat (2015): High-throughput, quantitative assessment of the effects of low-dose silica nanoparticles on lung cells: grasping complex toxicity with a great depth of field. BMC Genomics, 16: 315. doi: 10.1186/s12864-015-1521-5
[222]	M. S. Poulsen, T. Mose, L. L. Maroun, L. Mathiesen, L. E. Knudsen and E. Rytting (2015): Kinetics of silica nanoparticles in the human placenta. Nanotoxicology, 9 Suppl 1: 79-86. doi: 10.3109/17435390.2013.812259
[223]	S. Poussard, M. Decossas, O. Le Bihan, S. Mornet, G. Naudin and O. Lambert (2015): Internalization and fate of silica nanoparticles in C2C12 skeletal muscle cells: evidence of a beneficial effect on myoblast fusion. Int J Nanomedicine, 10: 1479-92. doi: 10.2147/IJN.S74158
[224]	G. Premshekharan, K. Nguyen, H. Zhang, H. J. Forman and V. J. Leppert (2017): Low dose inflammatory potential of silica particles in human-derived THP-1 macrophage cell culture studies - Mechanism and effects of particle size and iron. Chem Biol Interact, 272: 160-171. doi: 10.1016/j.cbi.2017.05.004
[225]	A. M. Prodan, C. S. Ciobanu, C. L. Popa, S. L. Iconaru and D. Predoi (2014): Toxicity evaluation following intratracheal instillation of iron oxide in a silica matrix in rats. Biomed Res Int, 2014: 134260. doi: 10.1155/2014/134260
[226]	S. Quignard, T. Coradin, J. J. Powell and R. Jugdaohsingh (2017): Silica nanoparticles as sources of silicic acid favoring wound healing in vitro. Colloids Surf B Biointerfaces, 155: 530-537. doi: 10.1016/j.colsurfb.2017.04.049
[227]	V. Rabolli, A. A. Badissi, R. Devosse, F. Uwambayinema, Y. Yakoub, M. Palmai-Pallag, A. Lebrun, V. De Gussem, I. Couillin, B. Ryffel, E. Marbaix, D. Lison and F. Huaux (2014): The alarmin IL-1alpha is a master cytokine in acute lung inflammation induced by silica micro- and nanoparticles. Part Fibre Toxicol, 11: 69. doi: 10.1186/s12989-014-0069-x
[228]	S. Rajiv, J. Jerobin, V. Saranya, M. Nainawat, A. Sharma, P. Makwana, C. Gayathri, L. Bharath, M. Singh, M. Kumar, A. Mukherjee and N. Chandrasekaran (2016): Comparative cytotoxicity and genotoxicity of cobalt (II, III) oxide, iron (III) oxide, silicon dioxide, and aluminum oxide nanoparticles on human lymphocytes in vitro. Hum Exp Toxicol, 35: 170-83. doi: 10.1177/0960327115579208
[229]	M. Ramasamy, M. Das, S. S. An and D. K. Yi (2014): Role of surface modification in zinc oxide nanoparticles and its toxicity assessment toward human dermal fibroblast cells. Int J Nanomedicine, 9: 3707-18. doi: 10.2147/IJN.S65086
[230]	R. Ramesh, P. Kavitha, N. Kanipandian, S. Arun, R. Thirumurugan and P. Subramanian (2013): Alteration of antioxidant enzymes and impairment of DNA in the SiO2 nanoparticles exposed zebra fish (Danio rerio). Environ Monit Assess, 185: 5873-81. doi: 10.1007/s10661-012-2991-4
[231]	F. Rancan, B. Nazemi, S. Rautenberg, M. Ryll, S. Hadam, Q. Gao, S. Hackbarth, S. F. Haag, C. Graf, E. Ruhl, U. Blume-Peytavi, J. Lademann, A. Vogt and M. C. Meinke (2014): Ultraviolet radiation and nanoparticle induced intracellular free radicals generation measured in human keratinocytes by electron paramagnetic resonance spectroscopy. Skin Res Technol, 20: 182-93. doi: 10.1111/srt.12104

[232]	L. Ren, J. Zhang, Y. Zou, L. Zhang, J. Wei, Z. Shi, Y. Li, C. Guo, Z. Sun and X. Zhou (2016): Silica nanoparticles induce reversible damage of spermatogenic cells via RIPK1 signal pathways in C57 mice. Int J Nanomedicine, 11: 2251-64. doi: 10.2147/IJN.S102268
[233]	F. Rios, A. Fernandez-Arteaga, M. Fernandez-Serrano, E. Jurado and M. Lechuga (2018): Silica micro- and nanoparticles reduce the toxicity of surfactant solutions. J Hazard Mater, 353: 436-443. doi: 10.1016/j.jhazmat.2018.04.040
[234]	M. Rothbauer, I. Praisler, D. Docter, R. H. Stauber and P. Ertl (2015): Microfluidic Impedimetric Cell Regeneration Assay to Monitor the Enhanced Cytotoxic Effect of Nanomaterial Perfusion. Biosensors (Basel), 5: 736-49. doi: 10.3390/bios5040736
[235]	H. J. Ryu, N. W. Seong, B. J. So, H. S. Seo, J. H. Kim, J. S. Hong, M. K. Park, M. S. Kim, Y. R. Kim, K. B. Cho, M. Y. Seo, M. K. Kim, E. H. Maeng and S. W. Son (2014): Evaluation of silica nanoparticle toxicity after topical exposure for 90 days. Int J Nanomedicine, 9 Suppl 2: 127-36. doi: 10.2147/IJN.S57929
[236]	R. Saborano, T. Wongpinyochit, J. D. Totten, B. F. Johnston, F. P. Seib and I. F. Duarte (2017): Metabolic Reprogramming of Macrophages Exposed to Silk, Poly(lactic-co-glycolic acid), and Silica Nanoparticles. Adv Healthc Mater, 6: doi: 10.1002/adhm.201601240
[237]	J. Saikia, M. Yazdimamaghani, S. P. Hadipour Moghaddam and H. Ghandehari (2016): Differential Protein Adsorption and Cellular Uptake of Silica Nanoparticles Based on Size and Porosity. ACS Appl Mater Interfaces, 8: 34820-34832. doi, https://www.ncbi.nlm.nih.gov/pubmed/27998138
[238]	K. Sakai-Kato, M. Hidaka, K. Un, T. Kawanishi and H. Okuda (2014): Physicochemical properties and in vitro intestinal permeability properties and intestinal cell toxicity of silica particles, performed in simulated gastrointestinal fluids. Biochim Biophys Acta, 1840: 1171-80. doi: 10.1016/j.bbagen.2013.12.014
[239]	A. Sanchez, J. L. Alvarez, K. Demydenko, C. Jung, Y. A. Alpizar, J. Alvarez-Collazo, S. M. Cokic, M. A. Valverde, P. H. Hoet and K. Talavera (2017): Silica nanoparticles inhibit the cation channel TRPV4 in airway epithelial cells. Part Fibre Toxicol, 14: 43. doi: 10.1186/s12989-017-0224-2
[240]	A. Scharf, K. H. Guhrs and A. von Mikecz (2016): Anti-amyloid compounds protect from silica nanoparticle-induced neurotoxicity in the nematode C. elegans. Nanotoxicology, 10: 426-35. doi: 10.3109/17435390.2015.1073399
[241]	I. Schremmer, A. Brik, D. G. Weber, N. Rosenkranz, A. Rostek, K. Loza, T. Bruning, G. Johnen, M. Epple, J. Bunger and G. A. Westphal (2016): Kinetics of chemotaxis, cytokine, and chemokine release of NR8383 macrophages after exposure to inflammatory and inert granular insoluble particles. Toxicol Lett, 263: 68-75. doi: 10.1016/j.toxlet.2016.08.014
[242]	C. Seidel, A. Kirsch, C. Fontana, A. Visvikis, A. Remy, L. Gate, C. Darne and Y. Guichard (2017): Epigenetic changes in the early stage of silica-induced cell transformation. Nanotoxicology, 11: 923-935. doi: 10.1080/17435390.2017.1382599
[243]	M. I. Setyawati, C. Y. Tay and D. T. Leong (2015): Mechanistic Investigation of the Biological Effects of SiO(2), TiO(2), and ZnO Nanoparticles on Intestinal Cells. Small, 11: 3458-68. doi: 10.1002/smll.201403232
[244]	B. Sharma, C. B. McLeland, T. M. Potter, S. T. Stern and P. P. Adiseshaiah (2018): Assessing NLRP3 Inflammasome Activation by Nanoparticles. Methods Mol Biol, 1682: 135-147. doi: 10.1007/978-1-4939-7352-1_12
[245]	K. H. Shim, K. H. Jeong, S. O. Bae, M. O. Kang, E. H. Maeng, C. S. Choi, Y. R. Kim, J. Hulme, E. K. Lee, M. K. Kim and S. S. An (2014): Assessment of ZnO and SiO2

	nanoparticle permeability through and toxicity to the blood-brain barrier using Evans blue and TEM. Int J Nanomedicine, 9 Suppl 2: 225-33. doi: 10.2147/IJN.S58205
[246]	J. H. Shin, K. Jeon, J. K. Kim, Y. Kim, M. S. Jo, J. S. Lee, J. E. Baek, H. S. Park, H. J. An, J. D. Park, K. Ahn, S. M. Oh and I. J. Yu (2017): Subacute inhalation toxicity study of synthetic amorphous silica nanoparticles in Sprague-Dawley rats. Inhal Toxicol, 29: 567-576. doi: 10.1080/08958378.2018.1426661
[247]	K. Shirasuna, F. Usui, T. Karasawa, H. Kimura, A. Kawashima, H. Mizukami, A. Ohkuchi, S. Nishimura, J. Sagara, T. Noda, K. Ozawa, S. Taniguchi and M. Takahashi (2015): Nanosilica-induced placental inflammation and pregnancy complications: Different roles of the inflammasome components NLRP3 and ASC. Nanotoxicology, 9: 554-67. doi: 10.3109/17435390.2014.956156
[248]	S. Siegrist, H. Kettiger, E. Fasler-Kan and J. Huwyler (2017): Selective stimulation of the JAK/STAT signaling pathway by silica nanoparticles in human endothelial cells. Toxicol In Vitro, 42: 308-318. doi: 10.1016/j.tiv.2017.05.002
[249]	T. Skuland, J. Ovrevik, M. Lag and M. Refsnes (2014): Role of size and surface area for pro-inflammatory responses to silica nanoparticles in epithelial lung cells: importance of exposure conditions. Toxicol In Vitro, 28: 146-55. doi: 10.1016/j.tiv.2013.10.018
[250]	T. Skuland, J. Ovrevik, M. Lag, P. Schwarze and M. Refsnes (2014): Silica nanoparticles induce cytokine responses in lung epithelial cells through activation of a p38/TACE/TGF-alpha/EGFR-pathway and NF-kappaBeta signalling. Toxicol Appl Pharmacol, 279: 76-86. doi: 10.1016/j.taap.2014.05.006
[251]	S. Smulders, K. Luyts, G. Brabants, K. V. Landuyt, C. Kirschhock, E. Smolders, L. Golanski, J. Vanoirbeek and P. H. Hoet (2014): Toxicity of nanoparticles embedded in paints compared with pristine nanoparticles in mice. Toxicol Sci, 141: 132-40. doi: 10.1093/toxsci/kfu112
[252]	M. J. Son, W. K. Kim, M. Kwak, K. J. Oh, W. S. Chang, J. K. Min, S. C. Lee, N. W. Song and K. H. Bae (2015): Silica nanoparticles inhibit brown adipocyte differentiation via regulation of p38 phosphorylation. Nanotechnology, 26: 435101. doi: 10.1088/0957-4484/26/43/435101
[253]	G. A. Sotiriou, C. Watson, K. M. Murdaugh, T. H. Darrah, G. Pyrgiotakis, A. Elder, J. D. Brain and P. Demokritou (2014): Engineering safer-by-design, transparent, silica-coated ZnO nanorods with reduced DNA damage potential. Environ Sci Nano, 1: 144-153. doi: 10.1039/C3EN00062A
[254]	K. Srikanth, T. Trindade, A. C. Duarte and E. Pereira (2017): Cytotoxicity and oxidative stress responses of silica-coated iron oxide nanoparticles in CHSE-214 cells. Environ Sci Pollut Res Int, 24: 2055-2064. doi: 10.1007/s11356-016-7870-z
[255]	M. S. Stan, I. Memet, C. Sima, T. Popescu, V. S. Teodorescu, A. Hermenean and A. Dinischiotu (2014): Si/SiO2 quantum dots cause cytotoxicity in lung cells through redox homeostasis imbalance. Chem Biol Interact, 220: 102-15. doi: 10.1016/j.cbi.2014.06.020
[256]	C. Strobel, M. Forster and I. Hilger (2014): Biocompatibility of cerium dioxide and silicon dioxide nanoparticles with endothelial cells. Beilstein J Nanotechnol, 5: 1795-807. doi: 10.3762/bjnano.5.190
[257]	B. Sun, S. Pokhrel, D. R. Dunphy, H. Zhang, Z. Ji, X. Wang, M. Wang, Y. P. Liao, C. H. Chang, J. Dong, R. Li, L. Madler, C. J. Brinker, A. E. Nel and T. Xia (2015): Reduction of Acute Inflammatory Effects of Fumed Silica Nanoparticles in the Lung by Adjusting Silanol Display through Calcination and Metal Doping. ACS Nano, 9: 9357-72. doi: 10.1021/acsnano.5b03443

[258]	M. P. Sutunkova, S. N. Solovyeva, B. A. Katsnelson, V. B. Gurvich, L. I. Privalova, I. A. Minigalieva, T. V. Slyshkina, I. E. Valamina, O. H. Makeyev, V. Y. Shur, I. V. Zubarev, D. K. Kuznetsov and E. V. Shishkina (2017): A paradoxical response of the rat organism to long-term inhalation of silica-containing submicron (predominantly nanoscale) particles of a collected industrial aerosol at realistic exposure levels. Toxicology, 384: 59-68. doi: 10.1016/j.tox.2017.04.010
[259]	K. Tabari, S. Hosseinpour, P. Parashos, P. Kardouni Khozestani and H. M. Rahimi (2017): Cytotoxicity of Selected Nanoparticles on Human Dental Pulp Stem Cells. Iran Endod J, 12: 137-142. doi: 10.22037/iej.2017.28
[260]	A. Tarantini, S. Huet, G. Jarry, R. Lanceleur, M. Poul, A. Tavares, N. Vital, H. Louro, M. Joao Silva and V. Fessard (2015): Genotoxicity of synthetic amorphous silica nanoparticles in rats following short-term exposure. Part 1: oral route. Environ Mol Mutagen, 56: 218-27. doi: 10.1002/em.21935
[261]	A. Tarantini, R. Lanceleur, A. Mourot, M. T. Lavault, G. Casterou, G. Jarry, K. Hogeveen and V. Fessard (2015): Toxicity, genotoxicity and proinflammatory effects of amorphous nanosilica in the human intestinal Caco-2 cell line. Toxicol In Vitro, 29: 398-407. doi: 10.1016/j.tiv.2014.10.023
[262]	A. M. Tavares, H. Louro, S. Antunes, S. Quarre, S. Simar, P. J. De Temmerman, E. Verleysen, J. Mast, K. A. Jensen, H. Norppa, F. Nesslany and M. J. Silva (2014): Genotoxicity evaluation of nanosized titanium dioxide, synthetic amorphous silica and multi-walled carbon nanotubes in human lymphocytes. Toxicol In Vitro, 28: 60-9. doi: 10.1016/j.tiv.2013.06.009
[263]	E. Tedesco, I. Micetic, S. G. Ciappellano, C. Micheletti, M. Venturini and F. Benetti (2015): Cytotoxicity and antibacterial activity of a new generation of nanoparticle-based consolidants for restoration and contribution to the safe-by-design implementation. Toxicol In Vitro, 29: 1736-44. doi: 10.1016/j.tiv.2015.07.002
[264]	M. Tsugita, N. Morimoto and M. Nakayama (2017): SiO2 and TiO2 nanoparticles synergistically trigger macrophage inflammatory responses. Part Fibre Toxicol, 14: 11. doi: 10.1186/s12989-017-0192-6
[265]	N. T. Vo, M. R. Bufalino, K. D. Hartlen, V. Kitaev and L. E. Lee (2014): Cytotoxicity evaluation of silica nanoparticles using fish cell lines. In Vitro Cell Dev Biol Anim, 50: 427-38. doi: 10.1007/s11626-013-9720-3
[266]	J. Wang, Y. Yu, K. Lu, M. Yang, Y. Li, X. Zhou and Z. Sun (2017): Silica nanoparticles induce autophagy dysfunction via lysosomal impairment and inhibition of autophagosome degradation in hepatocytes. Int J Nanomedicine, 12: 809-825. doi: 10.2147/IJN.S123596
[267]	K. Wang, J. Ma, M. He, G. Gao, H. Xu, J. Sang, Y. Wang, B. Zhao and D. Cui (2013): Toxicity assessments of near-infrared upconversion luminescent LaF3:Yb,Er in early development of zebrafish embryos. Theranostics, 3: 258-66. doi: 10.7150/thno.5701
[268]	W. Wang, Y. Li, X. Liu, M. Jin, H. Du, Y. Liu, P. Huang, X. Zhou, L. Yuan and Z. Sun (2013): Multinucleation and cell dysfunction induced by amorphous silica nanoparticles in an L-02 human hepatic cell line. Int J Nanomedicine, 8: 3533-41. doi: 10.2147/IJN.S46732
[269]	W. Wang, C. Zeng, Y. Feng, F. Zhou, F. Liao, Y. Liu, S. Feng and X. Wang (2018): The size-dependent effects of silica nanoparticles on endothelial cell apoptosis through activating the p53-caspase pathway. Environ Pollut, 233: 218-225. doi: 10.1016/j.envpol.2017.10.053
[270]	Z. Wang, C. Wang, S. Liu, W. He, L. Wang, J. Gan, Z. Huang, Z. Wang, H. Wei, J. Zhang and L. Dong (2017): Specifically Formed Corona on Silica Nanoparticles Enhances

	Transforming Growth Factor beta1 Activity in Triggering Lung Fibrosis. ACS Nano, 11: 1659-1672. doi: 10.1021/acsnano.6b07461
[271]	C. Watson, J. Ge, J. Cohen, G. Pyrgiotakis, B. P. Engelward and P. Demokritou (2014): High-throughput screening platform for engineered nanoparticle-mediated genotoxicity using CometChip technology. ACS Nano, 8: 2118-33. doi: 10.1021/nn404871p
[272]	J. L. Weaver, G. A. Tobin, T. Ingle, S. Bancos, D. Stevens, R. Rouse, K. E. Howard, D. Goodwin, A. Knapton, X. Li, K. Shea, S. Stewart, L. Xu, P. L. Goering, Q. Zhang, P. C. Howard, J. Collins, S. Khan, K. Sung and K. M. Tyner (2017): Evaluating the potential of gold, silver, and silica nanoparticles to saturate mononuclear phagocytic system tissues under repeat dosing conditions. Part Fibre Toxicol, 14: 25. doi: 10.1186/s12989-017-0206-4
[273]	F. Wei, Y. Wang, Z. Luo, Y. Li and Y. Duan (2017): New findings of silica nanoparticles induced ER autophagy in human colon cancer cell. Sci Rep, 7: 42591. doi: 10.1038/srep42591
[274]	M. N. Weitzmann, S. W. Ha, T. Vikulina, S. Roser-Page, J. K. Lee and G. R. Beck, Jr. (2015): Bioactive silica nanoparticles reverse age-associated bone loss in mice. Nanomedicine, 11: 959-967. doi: 10.1016/j.nano.2015.01.013
[275]	G. A. Westphal, I. Schremmer, A. Rostek, K. Loza, N. Rosenkranz, T. Bruning, M. Epple and J. Bunger (2015): Particle-induced cell migration assay (PICMA): A new in vitro assay for inflammatory particle effects based on permanent cell lines. Toxicol In Vitro, 29: 997-1005. doi: 10.1016/j.tiv.2015.04.005
[276]	M. Wiemann, A. Vennemann, U. G. Sauer, K. Wiench, L. Ma-Hock and R. Landsiedel (2016): An in vitro alveolar macrophage assay for predicting the short-term inhalation toxicity of nanomaterials. J Nanobiotechnology, 14: 16. doi: 10.1186/s12951-016-0164-2
[277]	J. W. Wills, N. Hondow, A. D. Thomas, K. E. Chapman, D. Fish, T. G. Maffeis, M. W. Penny, R. A. Brown, G. J. Jenkins, A. P. Brown, P. A. White and S. H. Doak (2016): Genetic toxicity assessment of engineered nanoparticles using a 3D in vitro skin model (EpiDerm). Part Fibre Toxicol, 13: 50. doi: 10.1186/s12989-016-0161-5
[278]	H. C. Winkler, J. Kornprobst, P. Wick, L. M. von Moos, I. Trantakis, E. M. Schraner, B. Bathke, H. Hochrein, M. Suter and H. Naegeli (2017): MyD88-dependent pro-interleukin-1beta induction in dendritic cells exposed to food-grade synthetic amorphous silica. Part Fibre Toxicol, 14: 21. doi: 10.1186/s12989-017-0202-8
[279]	A. Wittig, H. Gehrke, G. Del Favero, E. M. Fritz, M. Al-Rawi, S. Diabate, C. Weiss, H. Sami, M. Ogris and D. Marko (2017): Amorphous Silica Particles Relevant in Food Industry Influence Cellular Growth and Associated Signaling Pathways in Human Gastric Carcinoma Cells. Nanomaterials (Basel), 7: doi: 10.3390/nano7010018
[280]	A. Wolterbeek, T. Oosterwijk, S. Schneider, R. Landsiedel, D. de Groot, R. van Ee, M. Wouters and H. van de Sandt (2015): Oral two-generation reproduction toxicity study with NM-200 synthetic amorphous silica in Wistar rats. Reprod Toxicol, 56: 147-54. doi: 10.1016/j.reprotox.2015.03.006
[281]	J. Wu, Y. Shi, C. O. Asweto, L. Feng, X. Yang, Y. Zhang, H. Hu, J. Duan and Z. Sun (2016): Co-exposure to amorphous silica nanoparticles and benzo[a]pyrene at low level in human bronchial epithelial BEAS-2B cells. Environ Sci Pollut Res Int, 23: 23134-23144. doi: 10.1007/s11356-016-7559-3
[282]	Y. Xia, M. Li, T. Peng, W. Zhang, J. Xiong, Q. Hu, Z. Song and Q. Zheng (2013): In vitro cytotoxicity of fluorescent silica nanoparticles hybridized with aggregation-induced emission luminogens for living cell imaging. Int J Mol Sci, 14: 1080-92. doi: 10.3390/ijms14011080

[283]	B. Xu, Z. Mao, X. Ji, M. Yao, M. Chen, X. Zhang, B. Hang, Y. Liu, W. Tang, Q. Tang and Y. Xia (2015): miR-98 and its host gene Huwe1 target Caspase-3 in Silica nanoparticles-treated male germ cells. Sci Rep, 5: 12938. doi: 10.1038/srep12938
[284]	Y. Xu, N. Wang, Y. Yu, Y. Li, Y. B. Li, Y. B. Yu, X. Q. Zhou and Z. W. Sun (2014): Exposure to silica nanoparticles causes reversible damage of the spermatogenic process in mice. PLoS One, 9: e101572. doi: 10.1371/journal.pone.0101572
[285]	J. Y. Xue, X. Li, M. Z. Sun, Y. P. Wang, M. Wu, C. Y. Zhang, Y. N. Wang, B. Liu, Y. S. Zhang, X. Zhao and X. Z. Feng (2013): An assessment of the impact of SiO2 nanoparticles of different sizes on the rest/wake behavior and the developmental profile of zebrafish larvae. Small, 9: 3161-8. doi: 10.1002/smll.201300430
[286]	Y. Xue, Q. Chen, T. Ding and J. Sun (2014): SiO(2) nanoparticle-induced impairment of mitochondrial energy metabolism in hepatocytes directly and through a Kupffer cell-mediated pathway in vitro. Int J Nanomedicine, 9: 2891-903. doi: 10.2147/IJN.S60661
[287]	S. Yaman, U. Comelekoglu, E. Degirmenci, M. I. Karagul, S. Yalin, E. Balli, S. Yildirimcan, M. Yildirim, A. Doganer and K. Ocakoglu (2017): Effects of silica nanoparticles on isolated rat uterine smooth muscle. Drug Chem Toxicol, 1-11. doi: 10.1080/01480545.2017.1384005
[288]	E. J. Yang and I. H. Choi (2013): Immunostimulatory effects of silica nanoparticles in human monocytes. Immune Netw, 13: 94-101. doi: 10.4110/in.2013.13.3.94
[289]	H. Yang, Q. Y. Wu, C. S. Lao, M. Y. Li, Y. Gao, Y. Zheng and B. Shi (2016): Cytotoxicity and DNA damage in mouse macrophages exposed to silica nanoparticles. Genet Mol Res, 15: doi: 10.4238/gmr.15039005
[290]	H. Yang, Q. Y. Wu, M. Y. Li, C. S. Lao and Y. J. Zhang (2017): Pulmonary Toxicity in Rats Caused by Exposure to Intratracheal Instillation of SiO2 Nanoparticles. Biomed Environ Sci, 30: 264-279. doi: 10.3967/bes2017.036
[291]	H. Yang, Y. Zhang, W. Li, C. Lao, M. Li and Y. Zheng (2017): Altered microRNA expression profiles in lung damage induced by nanosized SiO2. Bioengineered, 8: 45-54. doi: 10.1080/21655979.2016.1227578
[292]	L. Yang, Q. Yan, J. Zhao, J. Li, X. Zong, L. Yang and Z. Wang (2013): The role of potassium channel in silica nanoparticle-induced inflammatory effect in human vascular endothelial cells in vitro. Toxicol Lett, 223: 16-24. doi: 10.1016/j.toxlet.2013.08.017
[293]	M. Yang, L. Jing, J. Wang, Y. Yu, L. Cao, L. Zhang, X. Zhou and Z. Sun (2016): Macrophages participate in local and systemic inflammation induced by amorphous silica nanoparticles through intratracheal instillation. Int J Nanomedicine, 11: 6217-6228. doi: 10.2147/IJN.S116492
[294]	X. Yang, C. He, J. Li, H. Chen, Q. Ma, X. Sui, S. Tian, M. Ying, Q. Zhang, Y. Luo, Z. Zhuang and J. Liu (2014): Uptake of silica nanoparticles: neurotoxicity and Alzheimer-like pathology in human SK-N-SH and mouse neuro2a neuroblastoma cells. Toxicol Lett, 229: 240-9. doi: 10.1016/j.toxlet.2014.05.009
[295]	Y. Yang, Y. Yu, J. Wang, Y. Li, Y. Li, J. Wei, T. Zheng, M. Jin and Z. Sun (2017): Silica nanoparticles induced intrinsic apoptosis in neuroblastoma SH-SY5Y cells via CytC/Apaf-1 pathway. Environ Toxicol Pharmacol, 52: 161-169. doi: 10.1016/j.etap.2017.01.010
[296]	Y. X. Yang, Z. M. Song, B. Cheng, K. Xiang, X. X. Chen, J. H. Liu, A. Cao, Y. Wang, Y. Liu and H. Wang (2014): Evaluation of the toxicity of food additive silica nanoparticles on gastrointestinal cells. J Appl Toxicol, 34: 424-35. doi: 10.1002/jat.2962

[297]	M. Yazdimamaghani, Z. B. Barber, S. P. Hadipour Moghaddam and H. Ghandehari (2018): Influence of Silica Nanoparticle Density and Flow Conditions on Sedimentation, Cell uptake and Cytotoxicity. Mol Pharm, doi: 10.1021/acs.molpharmaceut.8b00213
[298]	M. Yazdimamaghani, P. J. Moos and H. Ghandehari (2018): Global gene expression analysis of macrophage response induced by nonporous and porous silica nanoparticles. Nanomedicine, 14: 533-545. doi: 10.1016/j.nano.2017.11.021
[299]	H. Yi, Z. Wang, X. Li, M. Yin, L. Wang, A. Aldalbahi, N. N. El-Sayed, H. Wang, N. Chen, C. Fan and H. Song (2016): Silica Nanoparticles Target a Wnt Signal Transducer for Degradation and Impair Embryonic Development in Zebrafish. Theranostics, 6: 1810-20. doi: 10.7150/thno.16127
[300]	T. Yoshida, Y. Yoshioka, Y. Morishita, M. Aoyama, S. Tochigi, T. Hirai, K. Tanaka, K. Nagano, H. Kamada, S. Tsunoda, H. Nabeshi, T. Yoshikawa, K. Higashisaka and Y. Tsutsumi (2015): Protein corona changes mediated by surface modification of amorphous silica nanoparticles suppress acute toxicity and activation of intrinsic coagulation cascade in mice. Nanotechnology, 26: 245101. doi: 10.1088/0957-4484/26/24/245101
[301]	T. Yoshida, Y. Yoshioka, H. Takahashi, K. Misato, T. Mori, T. Hirai, K. Nagano, Y. Abe, Y. Mukai, H. Kamada, S. Tsunoda, H. Nabeshi, T. Yoshikawa, K. Higashisaka and Y. Tsutsumi (2014): Intestinal absorption and biological effects of orally administered amorphous silica particles. Nanoscale Res Lett, 9: 532. doi: 10.1186/1556-276X-9-532
[302]	T. Yoshida, Y. Yoshioka, S. Tochigi, T. Hirai, M. Uji, K. Ichihashi, K. Nagano, Y. Abe, H. Kamada, S. Tsunoda, H. Nabeshi, K. Higashisaka, T. Yoshikawa and Y. Tsutsumi (2013): Intranasal exposure to amorphous nanosilica particles could activate intrinsic coagulation cascade and platelets in mice. Part Fibre Toxicol, 10: 41. doi: 10.1186/1743-8977-10-41
[303]	C. Yu, Z. Zhou, J. Wang, J. Sun, W. Liu, Y. Sun, B. Kong, H. Yang and S. Yang (2015): In depth analysis of apoptosis induced by silica coated manganese oxide nanoparticles in vitro. J Hazard Mater, 283: 519-28. doi: 10.1016/j.jhazmat.2014.09.060
[304]	P. Yu, J. Li, J. Jiang, Z. Zhao, Z. Hui, J. Zhang, Y. Zheng, D. Ling, L. Wang, L. H. Jiang, J. Luo, X. Zhu and W. Yang (2015): A dual role of transient receptor potential melastatin 2 channel in cytotoxicity induced by silica nanoparticles. Sci Rep, 5: 18171. doi: 10.1038/srep18171
[305]	Y. Yu, J. Duan, W. Geng, Q. Li, L. Jiang, Y. Li, Y. Yu and Z. Sun (2015): Aberrant cytokinesis and cell fusion result in multinucleation in HepG2 cells exposed to silica nanoparticles. Chem Res Toxicol, 28: 490-500. doi: 10.1021/tx500473h
[306]	Y. Yu, J. Duan, Y. Li, Y. Li, L. Jing, M. Yang, J. Wang and Z. Sun (2017): Silica nanoparticles induce liver fibrosis via TGF-beta1/Smad3 pathway in ICR mice. Int J Nanomedicine, 12: 6045-6057. doi: 10.2147/IJN.S132304
[307]	Y. Yu, J. Duan, Y. Li, Y. Yu, M. Jin, C. Li, Y. Wang and Z. Sun (2015): Combined toxicity of amorphous silica nanoparticles and methylmercury to human lung epithelial cells. Ecotoxicol Environ Saf, 112: 144-52. doi: 10.1016/j.ecoenv.2014.10.026
[308]	Y. Yu, J. Duan, Y. Yu, Y. Li, X. Liu, X. Zhou, K. F. Ho, L. Tian and Z. Sun (2014): Silica nanoparticles induce autophagy and autophagic cell death in HepG2 cells triggered by reactive oxygen species. J Hazard Mater, 270: 176-86. doi: 10.1016/j.jhazmat.2014.01.028
[309]	Y. Yu, Y. Li, W. Wang, M. Jin, Z. Du, Y. Li, J. Duan, Y. Yu and Z. Sun (2013): Acute toxicity of amorphous silica nanoparticles in intravenously exposed ICR mice. PLoS One, 8: e61346. doi: 10.1371/journal.pone.0061346
[310]	J. W. Yun, S. H. Kim, J. R. You, W. H. Kim, J. J. Jang, S. K. Min, H. C. Kim, D. H. Chung, J. Jeong, B. C. Kang and J. H. Che (2015): Comparative toxicity of silicon dioxide, silver

	and iron oxide nanoparticles after repeated oral administration to rats. J Appl Toxicol, 35: 681-93. doi: 10.1002/jat.3125
[311]	Y. Zhang, Y. Lin, X. Li, L. Zhang, W. Pan, H. Zhu, Z. Xi and D. Yang (2017): Silica dioxide nanoparticles combined with cold exposure induce stronger systemic inflammatory response. Environ Sci Pollut Res Int, 24: 291-298. doi: 10.1007/s11356-016-7649-2
[312]	J. Zhu, L. Liao, L. Zhu, P. Zhang, K. Guo, J. Kong, C. Ji and B. Liu (2013): Size-dependent cellular uptake efficiency, mechanism, and cytotoxicity of silica nanoparticles toward HeLa cells. Talanta, 107: 408-15. doi: 10.1016/j.talanta.2013.01.037
[313]	X. Zhu, W. Cao, B. Chang, L. Zhang, P. Qiao, X. Li, L. Si, Y. Niu and Y. Song (2016): Polyacrylate/nanosilica causes pleural and pericardial effusion, and pulmonary fibrosis and granuloma in rats similar to those observed in exposed workers. Int J Nanomedicine, 11: 1593-605. doi: 10.2147/IJN.S102020
[314]	S. Zhuravskii, G. Yukina, O. Kulikova, A. Panevin, V. Tomson, D. Korolev and M. Galagudza (2016): Mast cell accumulation precedes tissue fibrosis induced by intravenously administered amorphous silica nanoparticles. Toxicol Mech Methods, 26: 260-9. doi: 10.3109/15376516.2016.1169341
[315]	Y. Zou, Q. Li, L. Jiang, C. Guo, Y. Li, Y. Yu, Y. Li, J. Duan and Z. Sun (2016): DNA Hypermethylation of CREB3L1 and Bcl-2 Associated with the Mitochondrial-Mediated Apoptosis via PI3K/Akt Pathway in Human BEAS-2B Cells Exposure to Silica Nanoparticles. PLoS One, 11: e0158475. doi: 10.1371/journal.pone.0158475
[316]	D. Zuo, Z. Duan, Y. Jia, T. Chu, Q. He, J. Yuan, W. Dai, Z. Li, L. Xing and Y. Wu (2016): Amphipathic silica nanoparticles induce cytotoxicity through oxidative stress mediated and p53 dependent apoptosis pathway in human liver cell line HL-7702 and rat liver cell line BRL-3A. Colloids Surf B Biointerfaces, 145: 232-240. doi: 10.1016/j.colsurfb.2016.05.006

2.1 List of publications with silica-coated materials

(Excerpt from complete list above)

Chia, SL et al. (2016). **Reducing ZnO nanoparticles toxicity through silica coating.** *Heliyon*, 2(10): e00177. **https://doi.org/10.1016/j.heliyon.2016.e00177**

Das, M et al. (2015). **Analyses of protein corona on bare and silica-coated gold nanorods against four mammalian cells.** *Int J Nanomedicine*, 10 1521-1545. **https://doi.org/10.2147/IJN.S76187**

Davidson, DC et al. (2016). **Direct stimulation of human fibroblasts by nCeO2 in vitro is attenuated with an amorphous silica coating.** *Part Fibre Toxicol*, 13(1): 23. **https://doi.org/10.1186/s12989-016-0134-8**

Ferchichi, S et al. (2016). **Evaluation of oxidative response and tissular damage in rat lungs exposed to silica-coated gold nanoparticles under static magnetic fields.** *Int J Nanomedicine*, 11 2711-2719. **https://doi.org/10.2147/IJN.S103140**

Fernandez-Bertolez, N et al. (2018). **Toxicological assessment of silica-coated iron oxide nanoparticles in human astrocytes.** *Food Chem Toxicol*, 118 13-23. **https://doi.org/10.1016/j.fct.2018.04.058**

Ha, SW et al. (2013). **Long-Term Monitoring of the Physicochemical Properties of Silica-Based Nanoparticles on the Rate of Endocytosis and Exocytosis and Consequences of Cell Division.** *Soft Mater*, 11(2): 195-203. **https://doi.org/10.1080/1539445X.2012.617641**

Konduru, NV et al. (2015). **Silica coating influences the corona and biokinetics of cerium oxide nanoparticles.** *Part Fibre Toxicol*, 12 31. https://doi.org/10.1186/s12989-015-0106-4
Konduru, NV et al. (2014). **Bioavailability, distribution and clearance of tracheally-instilled and gavaged uncoated or silica-coated zinc oxide nanoparticles.** *Part Fibre Toxicol*, 11 44. https://doi.org/10.1186/s12989-014-0044-6
Kononenko, V et al. (2017). **Harmful at non-cytotoxic concentrations: SiO2-SPIONs affect surfactant metabolism and lamellar body biogenesis in A549 human alveolar epithelial cells.** *Nanotoxicology*, 11(3): 419-429. https://doi.org/10.1080/17435390.2017.1309704
Leclerc, L et al. (2015). **Testicular biodistribution of silica-gold nanoparticles after intramuscular injection in mice.** *Biomed Microdevices*, 17(4): 66. https://doi.org/10.1007/s10544-015-9968-3
Leppanen, M et al. (2015). **Inhaled silica-coated TiO2 nanoparticles induced airway irritation, airflow limitation and inflammation in mice.** *Nanotoxicology*, 9(2): 210-218. https://doi.org/10.3109/17435390.2014.914260
Ma, J et al. (2015). **Effects of amorphous silica coating on cerium oxide nanoparticles induced pulmonary responses.** *Toxicol Appl Pharmacol*, 288(1): 63-73. https://doi.org/10.1016/j.taap.2015.07.012
Malvindi, MA et al. (2014). **Toxicity assessment of silica coated iron oxide nanoparticles and biocompatibility improvement by surface engineering.** *PLoS One*, 9(1): e85835. https://doi.org/10.1371/journal.pone.0085835
Phukan, G et al. (2016). **Silica-coated magnetic nanoparticles impair proteasome activity and increase the formation of cytoplasmic inclusion bodies in vitro.** *Sci Rep*, 6 29095. https://doi.org/10.1038/srep29095
Prodan, AM et al. (2014). **Toxicity evaluation following intratracheal instillation of iron oxide in a silica matrix in rats.** *Biomed Res Int*, 2014 134260. https://doi.org/10.1155/2014/134260
Ramasamy, M et al. (2014). **Role of surface modification in zinc oxide nanoparticles and its toxicity assessment toward human dermal fibroblast cells.** *Int J Nanomedicine*, 9 3707-3718. https://doi.org/10.2147/IJN.S65086
Sotiriou, GA et al. (2014). **Engineering safer-by-design, transparent, silica-coated ZnO nanorods with reduced DNA damage potential.** *Environ Sci Nano*, 1(2): 144-153. https://doi.org/10.1039/C3EN00062A
Srikanth, K et al. (2017). **Cytotoxicity and oxidative stress responses of silica-coated iron oxide nanoparticles in CHSE-214 cells.** *Environ Sci Pollut Res Int*, 24(2): 2055-2064. https://doi.org/10.1007/s11356-016-7870-z
Wang, K et al. (2013). **Toxicity assessments of near-infrared upconversion luminescent LaF3:Yb,Er in early development of zebrafish embryos.** *Theranostics*, 3(4): 258-266. https://doi.org/10.7150/thno.5701
Weitzmann, MN et al. (2015). **Bioactive silica nanoparticles reverse age-associated bone loss in mice.** *Nanomedicine*, 11(4): 959-967. https://doi.org/10.1016/j.nano.2015.01.013
Yu, C et al. (2015). **In depth analysis of apoptosis induced by silica coated manganese oxide nanoparticles in vitro.** *J Hazard Mater*, 283 519-528. https://doi.org/10.1016/j.jhazmat.2014.09.060

3 Datapoints for figures are taken from the following studies

(Numbers from the complete list above; number in red: high-quality studies)

Figure 2A and 2B. Values from: 1, 2, 5, 7, 9, 12, 13, 14, 15, 18, 24, 27, 30, 31, 33, 38, 41, 42, 43, 45, 52, 53, 61, 63, 64, 65, 68, 70, 72, 73, 76, 79, 80, 82, 84, 85, 87, 88, 89, 90, 91, 94, 97, 100, 100, 101, 103, 108, 109, 112, 113, 116, 118, 121, 122, 123, 124, 126, 127, 129, 130, 131, 134, 136, 145, 145,

149, 153, 159, 163, 164, 168, 169, 170, 182, 184, 187, 188, 189, 190, 195, 198, 199, 202, 203, 204, 207, 209, 211, 213, 214, 215, 222, 223, 224, 228, 231, 233, 234, 237, 238, 244, 248, 250, 252, 255, 259, 261, 263, 266, 268, 269, 271, 273, 277, 281, 282, 283, 286, 287, 288, 289, 292, 293, 294, 295, 296, 298, 299, 304, 307, 308, 312, 315, 316

Figure 3A and 3B. Values from: 2, 4, 8, 10, 13, 14, 19, 20, 24, 26, 30, 36, 48, 52, 53, 63, 69, 72, 73, 76, 80, 87, 90, 91, 100, 109, 113, 115, 116, 122, 123, 124, 127, 131, 134, 138, 144, 145, 148, 152, 153, 155, 156, 163, 168, 169, 176, 177, 182, 184, 186, 188, 189, 193, 196, 201, 206, 207, 211, 216, 228, 249, 256, 263, 276, 281, 282, 286, 295, 296, 297, 316.

Figure 4A and 4B. Values from: 2, 7, 8, 9, 10, 12, 24, 28, 36, 38, 41, 43, 52, 53, 62, 69, 70, 76, 85, 88, 90, 91, 92, 97, 109, 113, 115, 119, 123, 125, 126, 127, 145, 148, 149, 159, 163, 164, 168, 169, 171, 176, 184, 207, 211, 215, 216, 224, 228, 231, 248, 255, 261, 264, 268, 276, 281, 282, 294, 296, 298, 304, 307, 308, 316

Figure 5A and 5B. Values from: 1, 15, 19, 20, 24, 26, 28, 29, 41, 49, 56, 61, 62, 69, 72, 90, 106, 111, 122, 123, 134, 148, 152, 153, 178, 189, 198, 206, 207, 209, 224, 231, 236, 244, 249, 250, 257, 261, 264, 276, 278, 286, 288, 292
